# The recent outbreaks of human coronaviruses: A medicinal chemistry perspective

**DOI:** 10.1002/med.21724

**Published:** 2020-08-27

**Authors:** Thanigaimalai Pillaiyar, Lukas L. Wendt, Manoj Manickam, Maheswaran Easwaran

**Affiliations:** ^1^ PharmaCenter Bonn, Pharmaceutical Institute, Pharmaceutical & Medicinal Chemistry University of Bonn Bonn Germany; ^2^ Department of Chemistry PSG Institute of Technology and Applied Research Coimbatore Tamil Nadu India; ^3^ Department of Biomedical Engineering Sethu Institute of Technology Virudhunagar Tamilnadu India

**Keywords:** antivirals, COVID‐19, human coronavirus, main protease inhibitors, MERS‐CoV, SARS‐CoV‐1, SARS‐CoV‐2

## Abstract

Coronaviruses (CoVs) infect both humans and animals. In humans, CoVs can cause respiratory, kidney, heart, brain, and intestinal infections that can range from mild to lethal. Since the start of the 21st century, three β‐coronaviruses have crossed the species barrier to infect humans: severe‐acute respiratory syndrome (SARS)‐CoV‐1, Middle East respiratory syndrome (MERS)‐CoV, and SARS‐CoV‐2 (2019‐nCoV). These viruses are dangerous and can easily be transmitted from human to human. Therefore, the development of anticoronaviral therapies is urgently needed. However, to date, no approved vaccines or drugs against CoV infections are available. In this review, we focus on the medicinal chemistry efforts toward the development of antiviral agents against SARS‐CoV‐1, MERS‐CoV, SARS‐CoV‐2, targeting biochemical events important for viral replication and its life cycle. These targets include the spike glycoprotein and its host‐receptors for viral entry, proteases that are essential for cleaving polyproteins to produce functional proteins, and RNA‐dependent RNA polymerase for viral RNA replication.

**Abbreviations**
3CL^pro^
: 3C‐like protease
ACE: angiotensin‐converting enzyme
ACEi: ACE‐inhibitor
ADA: adenosine deaminase
Ang: angiotensin
ARB: angiotensin receptor blocker
ARDS: acute respiratory distress syndrome
CoV: coronaviruses
DPP: dipeptidyl peptidase
ESI‐MS: electrospray ionization mass spectrometry
HKU1: HCoV‐Hong Kong University 1
HR: heptad repeats
HTS: high throughput screening
ICTV: International Committee on Taxonomy of Viruses
IL: interleukin
kDa: kiloDalton
MERS: Middle East respiratory syndrome
MHV2: mouse hepatitis virus‐2
M^pro^
: main protease
NIH: National Institutes of Health
ORF: open reading frame
PCR: polymerase chain reaction
PL^pro^
: papain‐like protease
pp: polyprotein
RBD: receptor‐binding domain
RdRP: RNA‐dependent RNA polymerase
SARS: severe‐acute respiratory syndrome
TACE: TNF‐α converting enzyme
TGEV: transmissible gastroenteritis virus
TMPRSS: transmembrane serine protease

## 1 INTRODUCTION

**Figure 1:**
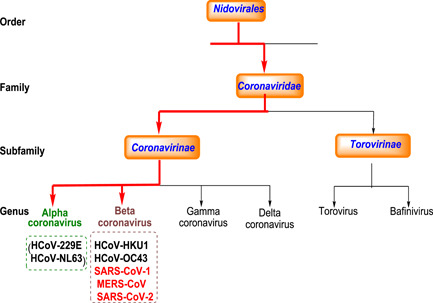
Schematic representation of the taxonomy of *Coronaviridae* (according to the International Committee on Taxonomy of Viruses). The seven human‐infecting coronaviruses belong to the α‐ or β‐coronavirus genus (highly infectious pathogens are highlighted red) [Color figure can be viewed at wileyonlinelibrary.com]

Coronaviruses (CoVs) infect humans and animals. In humans, CoVs cause primarily multiple respiratory and intestinal infections that can range from mild to lethal.^1^, ^2^, ^3^ According to the International Committee on Taxonomy of Viruses (ICTV), CoVs constitute the family *Coronaviridae* under the order *Nidovirales. Coronaviridae* comprise two subfamilies, *Torovirinae* and *Coronavirinae*, the latter being further divided into four main genera: α‐, β‐, γ‐, and δ‐coronaviruses (Figure 1).^4^ The history of human CoVs began in the 1930s, but only in the 1960s, the first human CoVs were identified in patients with mild respiratory infections, which were later named HCoV‐229E and HCoV‐OC43, belonging to α‐coronaviruses.^5^, ^6^, ^7^ Since then, virologists have discovered new viruses, studying their infection mechanisms, as well as their replication, and pathogenesis. This led to the identification of five novel CoVs belonging to β‐coronaviruses that have crossed the species barrier to infect humans: HCoV‐Hong Kong University 1 (HKU1), HCoV‐NL63, severe‐acute respiratory syndrome (SARS)‐CoV‐1, Middle East respiratory syndrome (MERS)‐CoV, and SARS‐CoV‐2 (COVID‐19).

The three last‐mentioned viruses are extremely dangerous because of their rapid transmission between humans. SARS‐CoV‐1, which emerged in 2002, affected 8096 in 32 countries, 774 of whom died (fatality rate 10%–15%).^8^ MERS‐CoV, which appeared in 2012, affected a total of 1841 individuals, 652 of whom died with the mortality rate of ~35% worldwide.^9^

The new coronavirus, known as SARS‐CoV‐2 or 2019‐nCoV has been identified as an etiological agent for the current epidemic with a contagious pneumonia‐like illness, spreading incredibly rapidly. As of July 15, 2020, the outbreak of SARS‐CoV‐2 has claimed more than 573 752 lives and infected more than 13 119 239 people around the planet.^10^ Public life has come to a halt, as many governments impose social distancing strategies and lockdown to prevent further spread of the virus. To date, no targeted therapeutics or vaccines are approved, and effective treatment options against any human‐infecting CoVs remain very limited.

### 1.1 Infection cycle of human CoVs and their druggable targets

Human‐infecting CoVs belonging to the α‐ and β‐CoV genera infect only mammals. According to the sequence database, all human CoVs have animal origins; HCoV‐NL63, HCoV‐229E, SARS‐CoV‐1, SARS‐CoV‐2, and MERS‐CoV are suggested to have originated from bats; HCoV‐OC43 and HKU1‐CoV likely originated from rodents.^11^, ^12^

CoVs are enveloped, single‐stranded, positive‐sense RNA viruses featuring the largest viral RNA genomes known to date, ranging roughly from 26 to 32 kilobases. The SARS‐CoV‐2 genome comprises ~30 000 nucleotides.^13^ For the virus to spread, the information of its structural and functional proteins must be replicated and packed into new virus particles. Since the virus lacks the necessary infrastructure for this process, it is entirely dependent on its host organism to translate its RNA into proteins and make more RNA copies.

To infect its desired host cell, the virus uses its many spike (S) glycoproteins protruding from its membrane.^13^ In general, the life cycle of CoVs can be classified into four main steps, including entry, replication, assembly, and release.

**Figure 2:**
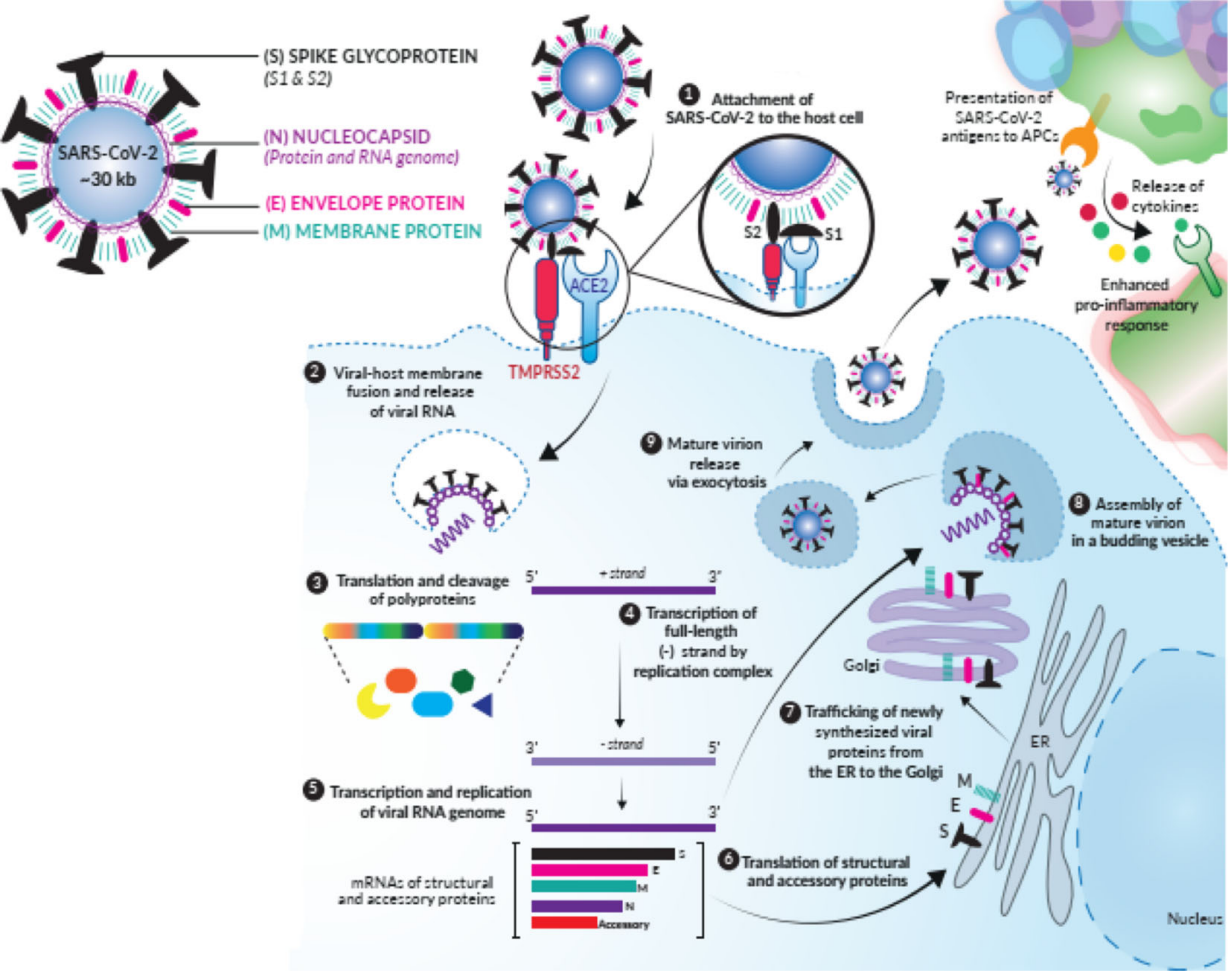
Infection cycle of coronaviruses, for example, SARS‐CoV‐2. The figure was adapted with permission from Invivogen (https://www.invivogen.com/spotlight-covid-19-infection). SARS‐CoV‐2, severe acute respiratory syndrome coronavirus 2 [Color figure can be viewed at wileyonlinelibrary.com]

The infection cycle of a CoV (Figure 2) begins with its entry. Using the S glycoprotein, it attaches itself to a surface receptor of the host cell. The host cell receptor and its distribution determine which tissues get infected. The specificity of the S protein to a particular receptor influences viral tropism. CoVs use different human receptors as points of entry: SARS‐CoV‐1, SARS‐CoV‐2, and HCoV‐NL63 use angiotensin‐converting enzyme 2 (ACE2);^14^, ^15^, ^16^ MERS‐CoV uses dipeptidyl peptidase‐4 (DPP4);^17^ CoV‐22E uses aminopeptidase N;^18^ and HCoV‐OC43 as well as HCoV‐HKUI use *O*‐acetylated sialic acid (see Table 1).^19^, ^20^

**Table 1:** Classification, discovery, cellular receptor, and natural intermediate host of the coronaviruses

HCoV genera	Coronaviruses	Discovery	Cellular receptor	Natural host(s)
α‐Coronaviruses	HCoV‐229E	1966	Human aminopeptidase N (CD13)	Bats
	HCoV‐NL63	2004	ACE2	Palm civets, bats
β‐Coronaviruses	HCoV‐OC43	1967	9‐*O*‐Acetylated sialic acid	Cattle
	HCoV‐HKU1	2005	9‐*O*‐Acetylated sialic acid	Mice
	SARS‐CoV‐1	2003	ACE2	Palm civets
	MERS‐CoV	2012	DPP4	Bats, camels
	SARS‐CoV‐2	2019	ACE2	Bats, (*?*)

*Note*: ? indicate other possible hosts of SARS‐CoV‐2, besides bats ‐ if they exist ‐ have not been conclusively identified yet.

When the spike protein attaches to its host cellular receptor, it is cleaved into two parts (S1 and S2) by extracellular proteases. While S1 remains attached to its target, S2 is further cleaved by the host cell's own transmembrane serine protease 2 (TMPRSS2). This process induces the fusion of the viral membrane with the host cell's membrane.^15^

Upon fusion, the contents of the virus particle are released into the host cell's cytoplasm. The virus's genomic positive‐sense RNA, which comprises two overlapping open reading frames (ORFs), ORF1a and ORF1b, is quickly translated into two polyproteins, pp1a and pp1ab. These proteins are the so‐called replicase‐transcriptase‐complex, because of their role in replication and further transcription. The newly formed polyproteins are immediately autocatalytically proteolyzed into smaller proteins by two viral proteases, 3C‐like protease (3CL^pro^), otherwise known as main protease (M^pro^), and papain‐like protease (PL^pro^).^21^, ^22^

The cleavage products include 16 nonstructural proteins (nsp) like the RNA‐dependent RNA polymerase (RdRP) that facilitates the production of antisense RNA, as well as 4 structural proteins like the S glycoprotein, envelope (E) proteins, membrane proteins (M), and nucleocapsid (N) proteins.^21^, ^22^, ^23^ Newly generated antisense RNA is used as a template for new copies of viral positive‐sense RNA as well as for the production of differently sized subgenomic mRNAs, which can be translated into new viral proteins at the endoplasmic reticulum. Finally, proteins and genomic RNA are assembled, packed into vesicles in the Golgi apparatus and exocytosed to the outside to repeat the process in surrounding cells.^23^

This process does not pass unnoticed by the host organism, as infected cells present viral structures on their surface. As a response, many defensive pathways are initiated, such as the production of different cytokines and chemokines like interleukin 1 (IL‐1), IL‐6, IL‐8, IL‐21, TNF‐β, and MCP‐1. The release of these mediators and their effector cells activate inflammatory mechanisms to destroy the intruder.^24^

The interruption of any stage of the viral life cycle can become an important therapeutic approach for treating CoV‐related diseases. A recent SARS‐CoV‐2‐human protein‐protein interaction analysis showed that SARS‐CoV‐2 contains approximately 66 druggable proteins, each of which has several ligand binding sites.^25^ The most interesting coronavirus proteins are the S glycoprotein, proteases M^pro^ and PL^pro^, RdRP, and helicase. In this review, we highlight these targets with potential therapeutic development against the highly dangerous pathogens SARS‐CoV‐1 and 2, and MERS‐CoV. Medicinal chemistry efforts toward the evolution of molecules with drug‐like properties is additionally discussed. In addition, broad‐spectrum antivirals targeting the major viruses are reviewed in detail, since they represent a highly promising strategy for treating these often fatal respiratory illnesses.

## 2 VIRUS ENTRY INHIBITORS

**Figure 3:**
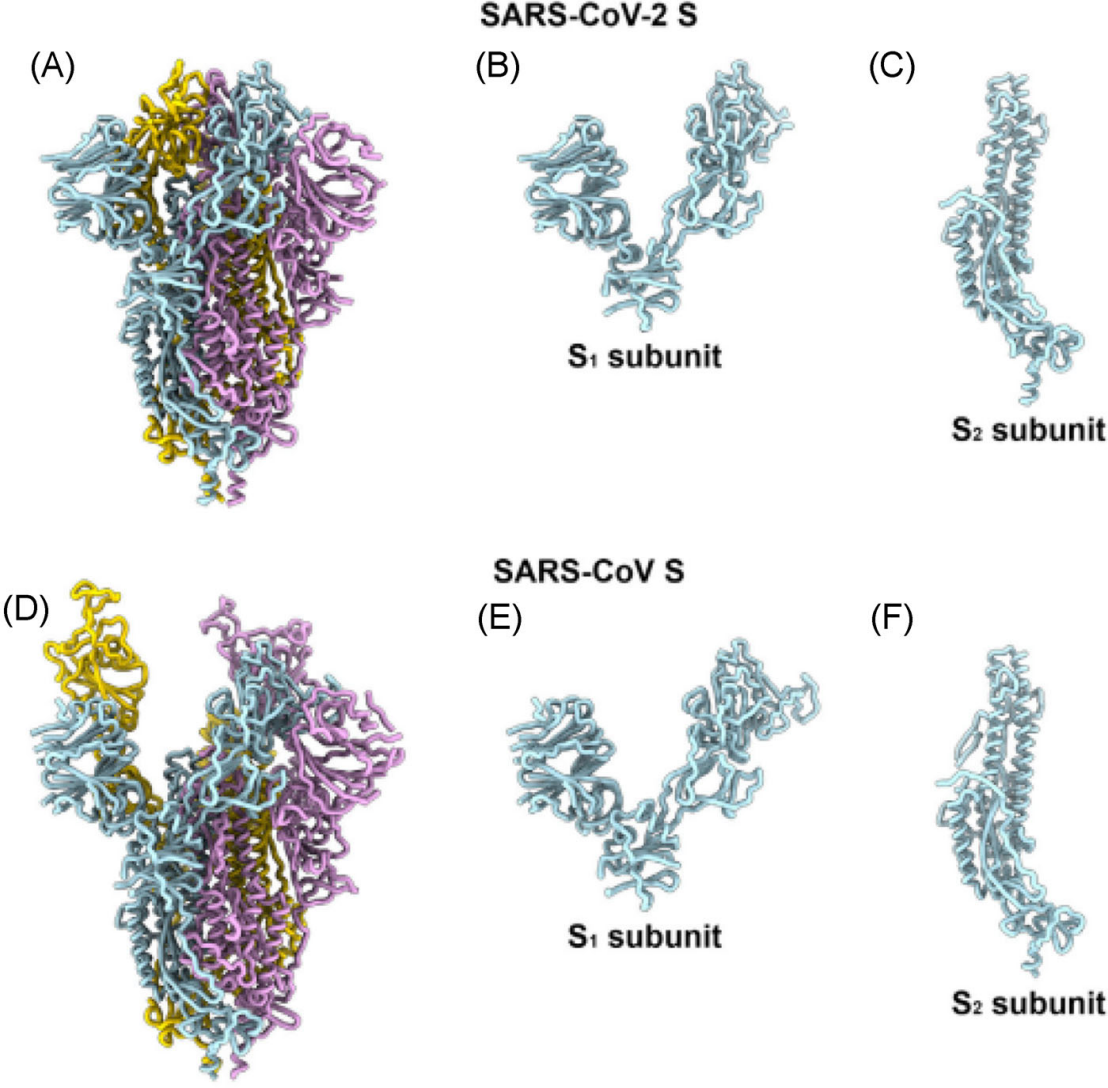
Comparison of the SARS‐CoV‐2 S and SARS‐CoV‐1 S structures: ribbon diagrams of the (A) SARS‐CoV‐2 S and (D) SARS‐CoV‐1 S [PDB 6NB6] ectodomain cryo‐EM structures. S1 subunits of (B) SARS‐CoV‐2 S and (E) SARS‐CoV‐1 S. S2 subunits of (C) SARS‐CoV‐2 S and (F) SARS‐CoV‐1 S.^28^ cryo‐EM, cryogenic electron microscopy; SARS‐CoV‐2, severe acute respiratory syndrome coronavirus 2 [Color figure can be viewed at wileyonlinelibrary.com]

Binding of spike protein (S) to its receptor represents the host's first confrontation with the virus and its life cycle, thus providing prophylactic intervention opportunities. The genome of SARS‐CoV‐2 has been recently determined to have an 80% identity to that of SARS‐CoV‐1 and 96% identity to the bat‐CoV RaTG13.^26^ The SARS‐CoV‐2 S protein shows nucleotide sequence identities of 75% or less to all other previously described CoVs. However, again, the new SARS‐CoV‐2 S protein shares a 93.1% identity to the S protein of RaTG13. SARS‐CoV‐2 spike (S) recognizes, with its receptor‐binding domain (RBD), the cellular ACE2 receptor with high affinity (*K*_d_, 14.7 nM)^27^ as judged by surface plasmin resonance spectrometry; and intervention at the RBD‐ACE2 interface can potentially disrupt infection efficiency. It was observed that the RBDs of the SARS‐CoV‐2‐ACE2 and SARS‐CoV‐1‐ACE2 complexes are quite similar (Figure 3).^29^

As mentioned previously, Zhang et al.^30^ determined the full‐length genome sequence of SARS‐CoV‐2 and revealed that the virus is remarkably similar (89.1% sequence identity) to a group of SARS‐like CoVs. Simultaneously, Shi et al.^26^ reported that SARS‐CoV‐2 shares 96% sequence identity at a whole‐genome level with a bat coronavirus—and importantly, like SARS‐CoV‐1, SARS‐CoV‐2 utilizes ACE2 receptor for viral entry. Recently, Yan et al. solved the cryo‐EM structure of full‐length human ACE2 bound to the RBD of SARS‐CoV‐2, providing important structural information for therapeutic intervention strategies.^29^

The sequence identity of the spike protein between SARS‐CoV‐1 (1273 aa) and SARS‐CoV‐2 (1253 aa) is 76%. The spike protein has two regions, S1 and S2. The S1 region of the SARS‐CoV‐1 has a RBD that forms high‐affinity interactions with ACE2. The prevailing understanding is that SARS‐CoV‐2 employs this RBD to enter its human host cell as well. Aligning the two different RBDs revealed a sequence identity of 73.5%. However, many nonconserved mutations that interact directly with ACE2 are located in the two structural regions.^31^ And both crystal and cryo‐EM structures of the SARS‐CoV‐1 spike‐ACE2 complex have shown that merely residues of regions 1 and 2 form hydrogen bonds and hydrophobic interactions with ACE2. The mutations in these two regions of SARS‐CoV‐2 will, therefore, likely reduce the number of those interactions.^32^

Studies also have shown that the RdRP, and the M^pro^ are highly conserved between SARS‐CoV‐2 and SARS‐CoV‐1.^33^, ^34^ Therefore, it is widely accepted that SARS‐CoV‐2 behaves similarly to SARS‐CoV‐1 with regard to viral entry and replication. Since the general genomic layout and replication kinetics are so conserved among MERS, SARS‐1, and SARS‐2 CoVs, investigating inhibitors of common structures is a logical step.

The inhibitory strength against viral enzyme was expressed as IC_50_, which is the concentration of the inhibitor needed to inhibit half of the enzyme activity in the tested condition. The *K*_i_ value is reflective of ligand‐binding affinity to the enzyme. The inhibitory activity for cell‐based bioassays is expressed as EC_50_, which is a half maximal effective concentration required to induce the biological response. The warhead group means a “reactive group” of the inhibitors that can form both covalent and noncovalent interactions with amino acids in the active site of the enzyme.

### 2.1 Targeting the RBD

Structural investigations of the RBD‐ACE2 complex provided information about essential residues for viral entry. Hsiang et al.^35^ reported a number of peptides that significantly blocked the interaction of the S protein with ACE2 with IC_50_ values as low as 1.88 nM. Michael et al. found charged residues between positions 22 and 57 crucial for SARS‐CoV‐1 viral entry. Based on this, they designed peptides P4 (IC_50_, 50 µM) and P5 (IC_50_, 6.0 µM) with significant inhibitory activity against SARS‐CoV‐1. The antiviral activity was further improved when they introduced the glycine binding linkage of peptide P4 (residues 22–47) with an ACE2‐derived peptide (residues 351–357) against a SARS‐CoV‐1 pseudovirus with an IC_50_ of 100 nM and devoid of cytotoxicity up to 200 µM.^36^ It is worth highlighting that a similar strategy could work for the new SARS‐CoV‐2. The recently solved cryo‐EM structure of SARS‐CoV‐2 in complex with human ACE2 can provide a structural rationale for the peptide design.^29^

For viral entry, MERS‐CoV uses its spike protein (S) to interact with the host‐receptor DPP4,^37^, ^38^, ^39^ also known as adenosine deaminase‐complexing protein‐2 or CD26.^37^ MERS‐CoV was also the first virus reported to use this particular path.^35^, ^37^ DPP4 is a type II transmembrane glycoprotein, that forms homodimers on the cell surface, and it is involved in the cleavage of dipeptides.^37^, ^40^ In humans, DPP4 is predominantly found on the bronchial epithelial and alveolar cells in the lower lungs.^40^, ^41^

MERS‐4 and MERS‐27 are monoclonal antibodies targeting the RBD of MERS‐CoV S that were discovered in a nonimmune yeast‐display scFv library screening. The more active MERS‐4 potently blocked the infection of DPP4‐expressing Huh‐7 cells with pseudotyped MERS‐CoV (IC_50_, 0.056 μg/mL). It also prevented MERS‐CoV‐induced cytopathogenic effects in MERS‐infected Vero E6 cells (IC_50_, 0.5 μg/mL).^42^

A heptad repeat (HR) is a repeating structural pattern of seven amino acids. A crucial membrane fusion framework of SARS‐CoV is the 6‐helix‐bundle (6‐HB) that is formed by HR1 and HR2 of the viral S protein. Enfuvirtide (T‐20) is an FDA approved HR2 peptide and the first HIV fusion inhibitor. It has opened up new avenues toward identifying and developing peptides as viral entry inhibitors. Such molecules represent a promising strategy against enveloped viruses with class 1 fusion proteins such as Nipah virus, Hendra virus, Ebola virus, and other paramyxoviruses, simian immunodeficiency virus, feline immunodeficiency virus, and respiratory syncytial virus.^43^, ^44^, ^45^, ^46^ The HR regions of SARS‐CoV‐1 and SARS‐CoV‐2 S protein share a high degree of conservation, and such fusion inhibitors have potential applications in preventing SARS‐CoV‐2 entry.

**Figure 4:**
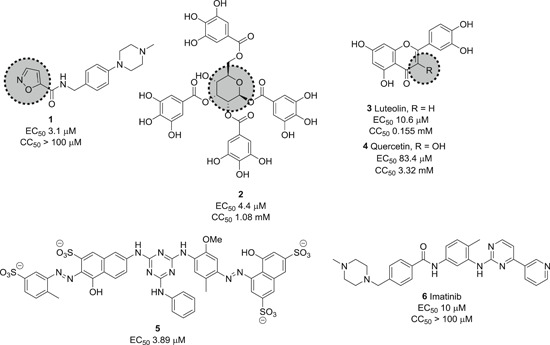
Inhibitors targeting the receptor‐binding domain

Small molecule entry inhibitors, on the other hand, are reported to target the RBD. Compared to peptides, proteins, and biologics, small molecules have several advantages due to lower production costs, improved pharmacokinetics, stability, and dosage accuracy. Sarafianos et al. identified the oxazole‐carboxamide derivative SSAA09E2 (**1**; Figure 4) as an entry inhibitor against SARS‐CoV‐1 by screening a chemical library composed of 3000 compounds.^47^ This inhibitor directly blocks ACE2 recognition by interfering with the RBD with an EC_50_ value of 3.1 µM and a 50% cytotoxic concentration (CC_50_) value of greater than 100 µM, not affecting ACE2 expression levels.^48^

Xu et al.^49^ identified two small molecules, TGG (**2**; Figure 4) and luteolin (**3**; Figure 4), that can bind avidly to the SARS‐CoV‐1 S2 protein and inhibit its entry into Vero E6 cells (EC_50_: 4.5 µM, 10.6 µM; respectively). Compounds **2** and **3** showed cytotoxicity (CC_50_) of 1.08 and 0.155 mM, and the selectivity index (SI) values of **2** and **3** were 240.0 and 14.62, respectively. Further studies regarding acute toxicity revealed that the 50% lethal doses of **2** and **3** were ~456 and 232 mg/kg, respectively. These results indicate that these small molecules could be used at relatively high concentrations in mice.^49^ Quercetin (**4**; Figure 4), an analog of **3**, also showed antiviral activity against SARS‐CoV‐1, with an EC_50_ value of 83.4 µM and a CC_50_ value of 3.32 mM.^50^

Ngai et al. reported ADS‐J1 (**5**; Figure 4) as a potential SARS‐CoV‐1 viral entry inhibitor with an EC_50_ of 3.89 µM. Molecular docking studies predicted that **5** can bind into a deep pocket of the SARS‐CoV‐1 S HR region and block viral entry into host cells.^51^ Imatinib (**6**; Figure 4), an Abelson kinase inhibitor, could inhibit CoV S protein‐induced fusion with an EC_50_ value of 10 µM and showed no cytotoxic effects in Vero cells up to 100 µM concentration.^52^, ^53^

### 2.2 Inhibitors targeting the cellular receptor

The genetic code of SARS‐CoV‐2 shares noticeable similarities with SARS‐CoV‐1, which caused the SARS epidemic in 2002.^26^, ^54^ More importantly, both viruses have identical mechanisms of infection. SARS‐CoV‐1 uses the host's ACE2 as a portal to infect cells, which has high expression in the vascular endothelium^55^ and the lung, particularly in type 2 alveolar epithelial cells.^56^ SARS‐CoV‐2 shares 76% of its spike (S) protein with SARS‐CoV‐1. Despite a few amino acid differences in its RBD compared to the SARS‐CoV‐1 S protein, the SARS‐CoV‐2 S protein binds to ACE2 with even greater affinity^27^ offering an explanation for its greater virulence and preference for the lung.

**Figure 5:**
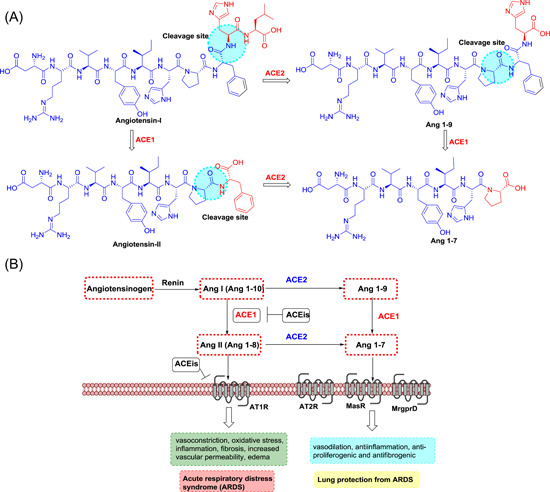
The roles of ACE1 and 2 in the renin‐angiotensin system. A, Chemical structures of angiotensin‐related peptides and B, Schematic diagram of roles of ACE1 and 2 in renin‐angiotensin system. ACE, angiotensin‐converting enzyme [Color figure can be viewed at wileyonlinelibrary.com]

ACE, a highly glycosylated type I integral membrane protein, is an essential component of the renin‐angiotensin (Ang) system, which controls blood pressure homeostasis. Both ACE1 and ACE2 cleave Ang peptides. However, they differ markedly: ACE1 cuts and converts the inactive decapeptide Ang I into the octapeptide Ang II by removing the dipeptide His‐Leu. This Ang II induces vaso‐ and bronchoconstriction, increased vascular permeability, inflammation, and fibrosis, thus promoting acute respiratory distress syndrome (ARDS) and lung failure in patients infected with SARS‐CoV‐1 or SARS‐CoV‐2^57^ (Figure 5). Therefore, ACE‐inhibitors (ACEis) and angiotensin II receptor blockers (ARBs) could block the disease‐propagating effect of Ang II.^58^, ^59^, ^60^

ACE2, on the other hand, is a zinc‐containing metalloenzyme, and shares merely 42% of its amino acid sequence with ACE1.^61^ It cleaves only one amino acid residue (Leu or Phe) from Ang I and Ang II, respectively, generating Ang (1–9) and Ang (1–7) (a vasodilator) (Figure 5). Thus, ACE2 has been considered a potential therapeutic target for cardiovascular diseases.

**Figure 6:**
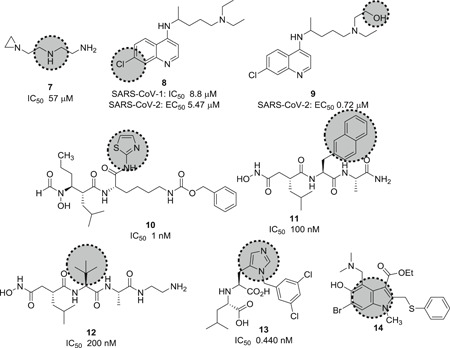
Inhibitors for SARS‐CoV‐1 and 2 targeting ACE2. ACE, angiotensin‐converting enzyme; SARS‐CoV, severe acute respiratory syndrome coronavirus

Virtual screening combined with a molecular docking approach targeting the ACE2 catalytic site with around 140 000 compounds led to the identification of inhibitor *N*‐(2‐aminoethyl)‐1 aziridine‐ethanamine (**7**; Figure 6) with an IC_50_ value of 57 µM and a *K*_i_ of 459 µM. However, no information about the cytotoxicity of this compound is available so far.^62^

Chloroquine (**8**; Figure 6) is a relatively safe, cheap, and effective medication for the treatment of malaria and amebiasis. Savarino et al.^63^ reported its antiviral effects. At a molecular level, it increases late endosomal and lysosomal pH, resulting in impaired liberation of virions from endosomes or lysosomes. The virus is therefore unable to release its genetic material into the cell and replicate.^64^, ^65^ Furthermore, they hypothesize that chloroquine might block the production of proinflammatory cytokines (such as IL‐6), thereby blocking the pathway that subsequently leads to ARDS.^63^

Chloroquine is reasonably active in vitro against SARS‐CoV‐1, MERS‐CoV, and SARS‐CoV‐2. It was found to inhibit SARS‐CoV‐2 with an EC_50_ value of 5.47 µM in vitro.^66^ Antiviral activity against SARS‐CoV‐1 was reported with an IC_50_ of 8.8 μM in Vero cells, but it is unclear how this translates into activity in respiratory epithelial cells and in vivo.^67^, ^68^ Mechanistic studies of chloroquine for SARS‐CoV‐1 infection revealed that it could also weaken the interaction between the RBD of SARS‐CoV‐1 and ACE2 by interfering with terminal glycosylation of ACE2, thereby reducing its affinity to SARS‐CoV‐1 S.^69^

During the SARS‐CoV‐2 pandemic, chloroquine has been recommended by Chinese, South Korean, and Italian health authorities for the experimental treatment of COVID‐19,^70^, ^71^ despite contraindications for patients with heart disease or diabetes.^72^ However, health experts and agencies like the US FDA and European Medicines Agency warned against broad uncontrolled use after reports of misuse of low‐quality versions of chloroquine phosphate intended for fish.

Hydroxychloroquine (**9**; Figure 6) is being studied as an experimental treatment for COVID‐19.^73^ However, the benefits of treatment with this drug are unclear.^74^

Hydroxychloroquine was found to inhibit SARS‐CoV‐2 with an EC_50_ value of 0.74 µM in vitro.^66^ Some studies imply synergistic effects of hydroxychloroquine and azithromycin. Azithromycin is active in vitro against Zika and Ebola virus^75^, ^76^ and can be used to guard against life‐threatening bacterial superinfections when administered to patients suffering from viral infections.^77^ A small study that compared hydroxychloroquine monotherapy and combination treatment with azithromycin found a significant advantage of the combination. While evaluating the efficacy of therapeutic intervention with hydroxychloroquine as monotherapy and its impact in combination with azithromycin, the number of patients testing negative in polymerase chain reaction (PCR) tests was substantially different in the two groups with 100% of patients cured (6 days post inclusion) in the combination arm of the study versus 57% in the monotherapy group. At the same time, 12% of patients in the control group receiving only standard care were cured.^78^, ^79^

The WHO declared on 18 March that chloroquine and its derivative hydroxychloroquine will be among the four medicines studied in the solidarity clinical trial^80^ for the treatment of COVID‐19. In April 2020, the US National Institutes of Health (NIH) also commenced a study with the drug for treating COVID‐19 patients.^81^

The recent clinical trial involving 96 032 patients with COVID‐19 concluded that it was unable to confirm a benefit of hydroxychloroquine or chloroquine, when used alone or in combination with a macrolide such as azithromycin (or clarithromycin).^82^ The study actually reported decreased survival rates for patients treated with each of these drug regimens. Additionally, patients had an increased risk of developing ventricular arrhythmia under treatment. However, still more evidence is needed to adequately assess the drugs' risks or benefits for the treatment or prevention of COVID‐19 (it is important to note that chloroquine and hydroxychloroquine are still considered safe treatment options in certain autoimmune diseases and malaria). Besides, the WHO announced the premature pause of its clinical trials using hydroxychloroquine as a safety precaution on 24 May 2020.

On a different note, it was found that ACE2 undergoes proteolytic shedding; releasing an enzymatic ectodomain during viral entry.^83^ A disintegrin and metalloproteinase (ADAM), also known as TNF‐α converting enzyme (TACE), assisted the shedding regulation of ACE2. Inhibition of this enzyme led to reduced shedding of ACE2. GW280264X (**10**; Figure 6) was found to be a specific inhibitor of ADAM‐induced shedding of ACE2 at 1 nM.^84^ Two TACE inhibitors, TAPI‐0 (**11**) and TAPI‐2 (**12**; Figure 6), reduced ACE2 shedding, with IC_50_ values of 100 and 200 nM, respectively.^83^

MLN‐4760 (**13**; Figure 6) inhibited the catalytic activity of ACE2 with an IC_50_ of around 440 pM.^85^ This is the most potent and selective small‐molecule inhibitor against soluble human ACE2 described to date, thus making it a very promising candidate for SARS‐CoV‐2 interference. It binds to the active site zinc and emulates the transition state peptide. However, no antiviral data for this compound is available at this time.

The interference of a virus‐host cell fusion, which is mediated by the viral S protein to its receptor ACE2 on host cells, may be a viable prevention strategy. Umifenovir (**14**; brand name Arbidol), a broad spectrum antiviral drug used against influenza, prevents viral entry by inhibiting virus‐host cell fusion.^86^ It is currently being investigated in a clinical trial for the treatment of SARS‐CoV‐2.^87^, ^88^

*Do ACEis or ARBs amplify SARS‐CoV‐2 pathogenicity and aggravate the clinical course of COVID‐19?* After ACE2 was recognized as the SARS‐CoV‐2 receptor,^14^, ^29^ speculations emerged about potentially negative consequences of ACEi or ARB therapy in COVID‐19 patients. This theory caused confusion in the public and alarmed patients taking these medicines. One report said that the expression of ACE2 was increased in patients with heart disease compared to healthy individuals. It was also insisted that ACE2 expression could be increased by taking ACEis and ARBs,^89^ although there is no supporting report of this happening in the lungs.

In another report, it was suggested that patients suffering from high blood pressure receiving “ACE2‐increasing drugs” have a higher risk for severe COVID‐19, since ACEis and ARBs could elevate levels of ACE2.^90^

A joint declaration by the presidents of the HFSA/ACC/AHA on 17 March 2020,^91^ followed by a similar statement of the European Medicines Agency,^92^ clarified that there was no scientific basis for stopping ACEi or ARB therapy.^93^, ^94^, ^95^ This was in accordance with the editors of the New England Journal of Medicine.^96^

In case of SARS‐CoV, the experimental data showed that such medications may be beneficial rather than damaging, which led to a new therapeutic approach for lung diseases.^97^

### 2.3 Proteolytic processing inhibitors

CoVs enter the host cells via both clathrin (endosomal) and nonclathrin pathways (nonendosomal); however, both pathways are dependent upon receptor binding.^98^, ^99^

The clathrin‐mediated pathway involves the binding of CoV S protein to the host receptor followed by the internalization of vesicles that maturate to late endosomes. Acidification of the endosome promotes the H^+^‐dependent activation of cellular cathepsin L proteinase in late endosomes and lysosomes, which cleaves and activates the S protein, thus initiating viral fusion. Recent research shows that in addition to ACE2 SARS‐CoV‐2 can also use the host cell receptor CD147 to gain access into host cells.^100^

Membrane fusion is also the crucial step for the CoV life cycle in the nonclathrin/endosomal route, in which host proteases such as cathepsin L, TMPRSS2, and TMPRSS11D (airway trypsin‐like protease) cut the S protein at the S1/S2 cleavage site to activate the S protein for membrane fusion.^101^ Interference with this process by targeting these proteases could become an attractive strategy for combating CoV infections. A recent study confirms the role of TMPRSS2 for the viral life cycle in SARS‐CoV‐2‐infected VeroE6 cells.^5^ Furin (a serine endoprotease) activates MERS‐CoV to initiate the nonclathrin mediated membrane fusion event.^102^

**Figure 7:**
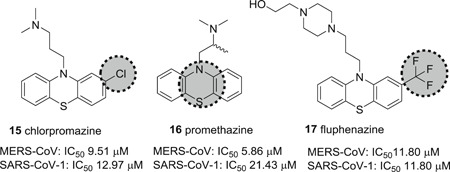
Neurotransmitter inhibitors targeting clathrin/nonclathrin pathways

The neurotransmitter receptor blockers chlorpromazine (**15**), promethazine (**16**), and fluphenazine (**17**; Figure 7), were reported to inhibit MERS‐CoV and SARS‐CoV‐1 most probably by impeding S protein‐induced fusion.^103^ Chlorpromazine, a clathrin‐mediated viral entry inhibitor, was already described to inhibit human CoV‐229E, hepatitis C virus, infectious bronchitis virus, as well as mouse hepatitis virus‐2 (MHV2).^104^, ^105^, ^106^, ^107^, ^108^

**Figure 8:**
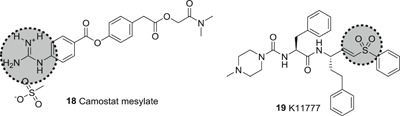
Inhibitors targeting TMPRSS2. TMPRSS, transmembrane serine protease

Matsuyama et al. identified the commercially available serine protease inhibitor camostat (**18**; Figure 8) to be a SARS‐CoV‐1 inhibitor, blocking TMPRSS2 activity at 10 µM. However, at a higher concentration (100 µM), inhibition of viral entry via SARS‐CoV‐1 S protein‐mediated cell fusion never exceeded 65% (inhibition efficiency), indicating that 35% of entry events take place via the endosomal cathepsin pathway. Interestingly, treatment with a combination of EST (a cathepsin inhibitor) and **18** resulted in remarkably blocked infection (>95%) activity of pseudotyped viruses.^109^

A similar approach has been investigated to prevent viral entry of SARS‐CoV‐2. Pöhlmann et al. reported the attainment of full inhibition efficiency with a combination of both **18** and E‐64d (a cathepsin inhibitor). Both studies indicate that SARS‐CoV‐1 and 2 enter cells in a similar manner showing the potential of **18** as a candidate for further development.^15^

Recently, K11777 (**19**; Figure 8), a cysteine protease inhibitor, was shown in tissue cultures to inhibit SARS‐CoV‐1 and MERS‐CoV replication in the subnanomolar range.^110^, ^111^ Future tissue culture and animal model studies should be conducted to clarify, whether its antiviral activity is mediated by targeting TMPRSS2.

Teicoplanin is a glycopeptide antibiotic used to prevent infections with Gram‐positive bacteria like methicillin‐resistant *Staphylococcus aureus* and *Enterococcus faecalis*. It was found that teicoplanin inhibits the entry of SARS‐CoV‐1, MERS‐CoV, and Ebola virus by specifically targeting cathepsin L.^112^ This knowledge has also been used to block the entry of new SARS‐CoV‐2 pseudoviruses with an IC_50_ value of 1.66 µM. Therefore, teicoplanin could be considered a potential candidate for the treatment of COVID‐19.^113^

### 2.4 Small‐molecules as cathepsin L inhibitors

**Figure 9:**
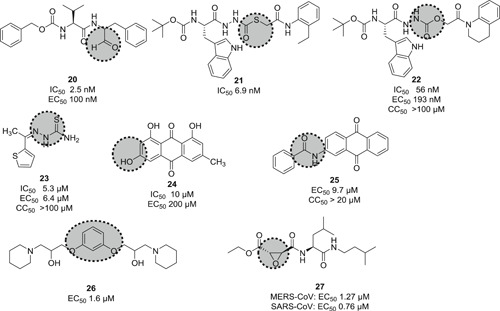
Cathepsin L inhibitors with antiviral activity

Human cathepsin L is a cysteine endopeptidase and plays a key role for infection efficiency by activation of the S protein into a fusogenic state to escape the late endosomes. Targeting this protease with small molecules could interfere with virus‐cell entry and therefore be a possible intervention strategy for CoV infection.^114^ Bates et al. identified MDL28170 (**20**; Figure 9) as an antiviral compound that specifically inhibited cathepsin‐L‐mediated substrate cleavage, with an IC_50_ value of 2.5 nM and EC_50_ value in the range of 100 nM. However, despite its potent inhibitory activity, no cytotoxicity data for **20** is currently available.^115^

Diamond et al. reported CID 16725315 (**21**) and CID 23631927 (**22**; Figure 9) as viral entry inhibitors of SARS‐CoV in a cathepsin L inhibition assay. Compound **21** could block cathepsin L with an IC_50_ value of 6.9 nM, while **22** showed slightly weaker potency (IC_50_, 56 nM). Compound **22** was also found to inhibit Ebola virus infection (EC_50_, 193 nM) of human embryonic kidney 293T cells. This compound did not show any sign of toxicity to human aortic endothelial cells up to 100 µM. This data offers a new promising point for the treatment of SARS and Ebola virus infections.^116^

Screening of ~14 000 compounds in a cell‐based assay resulted in the identification of SSAA09E1 (**23**; Figure 9) as inhibitor of cathepsin L proteinase, with an IC_50_ value of 5.33 µM. In a pseudotype‐based assay in 293T cells, the EC_50_ value of **23** was around 6.4 µM, and no cytotoxicity was detected below 100 µM.^48^

Phenotypic screening approaches led to the identification of several viral entry inhibitors. This approach has the advantage of finding cellular‐active compounds, providing information on drug solubility and cell uptake.^117^ On the other hand, it is limited in terms of capacity compared to in silico target‐based screening. Hsiang et al. identified emodin (**24**; Figure 9), the active component from *Polygonum multiflorum* and *Rheum officinale*, could block the interaction of S protein with ACE2, with an IC_50_ value of 10 µM and an EC_50_ value of 200 µM in an S protein‐pseudotyped retrovirus assay using Vero E6 cells. However, the mechanism of action of this compound still needs to be determined.^118^ Sarafianos et al.^48^ found that SSAA09E3 (**25**), a benzamide derivative of **24**, could prevent virus‐cell membrane fusion in pseudotype‐based and antiviral‐based assays, with an EC_50_ value of 9.7 µM, but a CC_50_ value of 20 µM indicates additional unknown cellular targets.

VE607 (**26**) was identified among 50 240 structurally diverse small molecules to specifically inhibit SARS‐CoV‐1 entry into cells using a phenotype‐based screening. Its EC_50_ value was reported at 3.0 µM and it inhibited SARS‐CoV‐1 plaque formation with an EC_50_ of 1.6 µM.^119^ Cathepsin inhibitor E‐64‐D (**27**) blocked MERS‐CoV and SARS‐CoV‐1 infection as well.^120^, ^121^

## 3 PROTEASES AS A DRUG TARGETS

Papain‐like protease (PL^pro^), and, predominantly, M^pro^ are required for the proteolytic cleavage of polyproteins produced by the virus. Together they produce 16 nsp that are involved in viral replication and transcription.^122^ PL^pro^ is responsible for cleavage at the first three positions of its polyprotein to produce three nsp, while M^pro^ cleaves at no less than 11 conserved sites, releasing nsp4 to nsp16. M^pro^‐mediated cleavage generates functional proteins like RdRP, RNA binding proteins, exoribonuclease, helicase, and methyltransferase.^123^ The indispensable role of M^pro^ for the viral life cycle and infection process makes M^pro^ an ideal target for anti‐coronaviral therapy.

### 3.1 M^pro^ inhibitors

#### 3.1.1 Structure and function of CoV M^pro^

M^pro^ is a homodimeric cysteine protease. The SARS‐CoV‐1 M^pro^ consists of three domains: I (residues 8–101), and II (residues 102–184), which are β‐barrel domains that shape the chymotrypsin‐like structure, while domain III (residues 201–306) is made up by α‐helices.^124^ The CoV M^pro^ active site uses a catalytic dyad (Cys145‐His41), in which cysteine acts as the nucleophile in the proteolysis while histidine behaves as general acid‐base. The peptide substrate or inhibitor binds in a cleft between domains I and II.^125^

As far as the development of new therapeutics against SARS‐ and MERS‐CoV infection is concerned, efforts have mainly focused on protease inhibitors. These enzymes are highly attractive drug targets because they are so essential to the virus. Peptides, peptidomimetics, and even small molecules can inhibit them, which leads to markedly reduced viral transmission and pathogenicity. Although most of the reported molecules display only weak anti‐CoV activity, several of studies elucidated structure–activity relationships that can be used to further improve their activity.^100^, ^126^, ^127^, ^128^

#### 3.1.2 Substrate‐derived M^pro^ inhibitors

**Figure 10:**
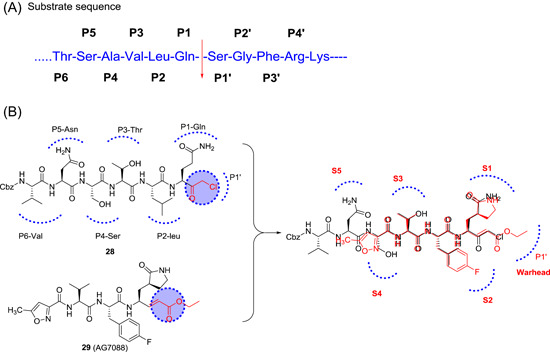
A, SARS‐CoV‐1 M^pro^ partial substrate sequence. B, (Overlay) structures of SARS‐CoV M^pro^ inhibitors. M^pro^, main protease; SARS‐CoV, severe acute respiratory syndrome coronavirus [Color figure can be viewed at wileyonlinelibrary.com]

To date, no approved drugs or vaccines are available for treating a coronavirus infection. In a race to identify chemotherapeutic options, various approaches, such as chemical synthesis, testing of natural products, and virtual screening of compound libraries, have been used. The systematic design of inhibitors of CoV M^pro^ was essentially based on the enzyme's substrate. In general, a substrate can be transformed into a good inhibitor by modifying part of its sequence such that it binds to the catalytic cysteine in either a reversible or an irreversible manner. Peptide inhibitors are designed by attaching a reactive group (also known as warhead group) to peptides that mimic the natural substrate. The partial peptide substrate sequence for SARS‐CoV‐1 M^pro^ is mentioned in Figure 10, indicating the specific subsite of each amino acid residue.

#### 3.1.3 Inhibitors with Michael acceptor as a warhead group

The disclosure of the first crystal structure of the SARS‐CoV‐1 M^pro^ in complex with a peptidic inhibitor Cbz‐Val‐Asn‐Ser‐Thr‐Leu‐Gln‐chloromethyl ketone (also known as hexapeptide chloromethyl ketone; **28**)^125^ provided clues for the substrate‐based design. Although it is a substrate analog for the porcine transmissible gastroenteritis CoV (TGEV) M^pro^, it offers a structural explanation for the P1‐Gln entering into the specific subsite S1 pocket and decreased P2‐leucine specificity in the hydrophobic S2 site of SARS‐CoV‐1 M^pro^. Additionally, rupintrivir (**29**; AG7088), a peptidomimetic inhibitor of human rhinovirus 3C protease is oriented similar to inhibitor **28** in the binding pocket of TGEV M^pro^.^129^ These two molecules became prototype compounds for the development of SARS‐CoV‐1 M^pro^ inhibitors.

**Figure 11:**
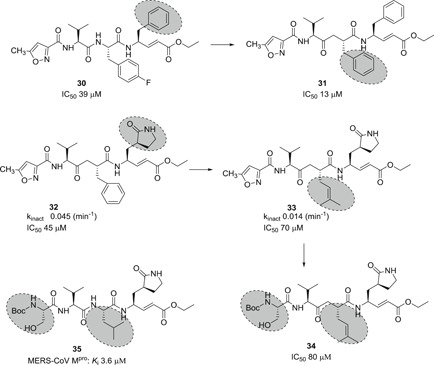
SARS‐CoV M^pro^ inhibitors containing Michael acceptor as a warhead group. M^pro^, main protease; SARS‐CoV, severe acute respiratory syndrome coronavirus

Compound **29** was only weakly active against SARS‐CoV‐1 M^pro^ (IC_50_, 800 µM) also in cellular antiviral assays.^130^ However, systematic structural modifications led to a series of analogs that show moderate to good activity.^131^ For example, compound **30** (Figure 11), in which the P1‐lactam was replaced by a phenyl ring, showed moderate activity. Compound **31**, in which the larger P2 *p*‐fluorophenyl was replaced with a phenyl group, was even more effective. By taking **29** as a lead, Ghosh et al. designed new molecules mainly focusing on the replacement of the large P2 *p*‐fluorobenzyl group. Two of the resulting structures with P2‐benzyl (**32**) and prenyl (**33**) moieties showed decent inhibitory potencies at both enzymatic (*K*_inact_, 0.014 and 0.045 min^−1^, respectively) and cell‐based (IC_50_, 45 and 70 µM) assays.^132^ Besides, no cytotoxicity was observed for these compounds up to 100 µM concentration. However, **32** and **33** were inactive at MERS‐CoV M^pro^.^133^ The same research group further modified the molecule with the introduction of P4 Boc‐serine, to establish additional hydrogen bond interactions as described in compound **34** (IC_50_, 75 µM). Unfortunately, the activity of the resulting compound was not improved. Further modification of the isobutyl group in compound **34** to isoprenyl group in compound **35** displayed potent activity with *K*_i_ = 3.6 µM (Figure 11).^14^

**Figure 12:**
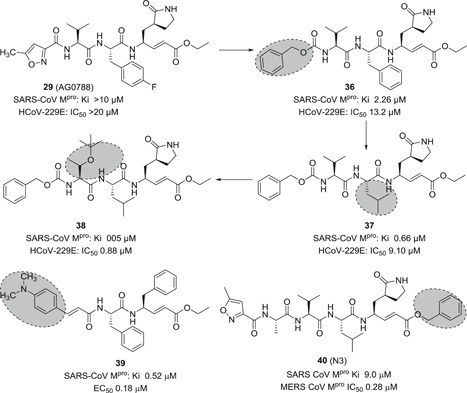
Broad‐spectral antiviral compounds containing a Michael acceptor

On the other hand, Yang et al.^134^ reported a series of peptide inhibitors with a greater inhibitory potency. In general, they systematically changed the backbone of inhibitor **29**. As a result, they were able to identify more specific residues for each subsite (compounds **36**–**38**; Figure 12): At first, the P1‐lactam ring was identified as a more specific moiety for the S1‐site, forming multiple hydrogen‐bond interactions with the enzyme as can be seen in the crystal structure (**36**); P2‐leucine showed a fourfold increased inhibitory activity when compared to the P2‐phenylalanine or ‐4‐fluorophenylalanine (**37**). A lipophilic *tert*‐butyl residue was recognized to be a better P3‐moiety than the P3‐valine (**38**). Finally, the replacement of P4‐methylisoxazole with a benzyloxy group was the best option for activity enhancement (compare **29** vs **36**). They all showed moderate to high antiviral activity against HCoV‐229E in cell‐based assays.

Shie et al.^131^ reported another series of peptide inhibitors with comparatively reduced molecular weight to increase drug‐like properties. These pseudo‐C2‐symmetric inhibitors consist of a Phe‐Phe‐dipeptidic α,β‐unsaturated ester. One of these inhibitors (**39**) had an outstanding inhibitory activity with an EC_50_ value of 0.52 µM (see Figure 12). Besides, it displayed remarkable antiviral activity with an EC_50_ value of 0.18 µM. Structurally, the presence of 4‐dimethylamine on the phenyl ring was found to be crucial for activity enhancement.

Another peptidic drug with a Michael acceptor was N3 (**40**), which was reported to inhibit SARS‐CoV‐1 3CL^pro^ (*K*_i_, 9.0 µM) by Yang et al. It was observed to be a broad‐spectrum antiviral compound, also inhibiting other CoVs, such as MERS‐CoV M^pro^ (IC_50_, 0.28µM),^135^ HCoV‐229E, HCoV‐NL63, and HCoV‐HKU1 M^pro^.^135^, ^136^, ^137^, ^138^ It has also exhibited high antiviral activity in an animal model of infectious bronchitis virus.^137^ The CC_50_ of **40** is greater than 133 μM.

**Figure 13:**
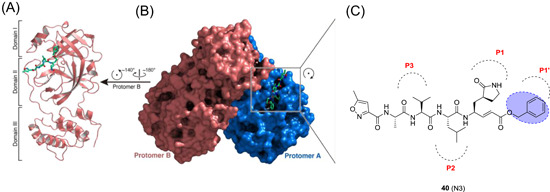
The crystal structure of COVID‐19 virus M^pro^ in complex with N3. (A) Representation of the dimeric M^pro^‐inhibitor complex. (B) Surface representation of the homodimer of M^pro^. Protomer A (blue), protomer B (salmon), compound N3 is presented as green sticks. (C) Schematic view of compound N3 (**40**) in the substrate‐binding pocket.^143^ M^pro^, main protease [Color figure can be viewed at wileyonlinelibrary.com]

SARS‐CoV‐2 shares only 82% of its genome with its relative SARS‐CoV‐1. However, essential viral enzymes of both species show sequence similarities of greater than 90%.^137^, ^139^, ^140^, ^141^, ^142^ SARS‐CoV‐2 3CL^pro^ is highly similar to SARS‐CoV‐1 3CL^pro^, sharing 96% of its sequence. Therefore, one could expect that SARS‐CoV‐1 M^pro^ inhibitors are active against SARS‐CoV‐2 M^pro^. Compound **40** was found to be active against SARS‐CoV‐2 M^pro^ and its value of kobs/[I] for the COVID‐19 virus M^pro^ was determined to be 11 300 ± 880 M^−1^·s^−1^.^143^ Peptide N3 was co‐crystalized with SARS‐CoV‐1 M^pro^ at 2.1 Å resolution (see Figure 13). Its binding mode to SARS‐CoV‐2 M^pro^ is highly similar to that of other CoV main proteases. Some key features include the Cys‐His catalytic dyad and the substrate‐binding pocket situated in a gap between domain I and II.

**Figure 14:**
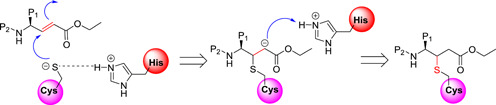
Mechanism of inhibitors with Michael acceptor group [Color figure can be viewed at wileyonlinelibrary.com]

In general, inhibitors possessing a Michael acceptor group as a warhead moiety could form an irreversible (covalent) bond with the catalytic cysteine residue in the following manner (Figure 14): First, the cysteine residue undergoes 1,4‐addition at the inhibitor's Michael acceptor group (warhead). Rapid protonation of the α‐carbanion from His‐H^+^ leads to the covalent bond formation between the warhead of the inhibitor and the cysteine residue.

#### 3.1.4 Inhibitors with aldehyde as a warhead group

Although the above‐described inhibitors with 1,4‐Michael acceptors (e.g., α,β‐vinyl ethyl ester, –CH═CH–C(O)–OEt) showed enzymatic or cell‐based in‐vitro activities, they can be cleaved to their carboxylic acids by plasma esterases; for instance, AG7088 (**29**) was inactive in the plasma of rodents and rabbits.^144^, ^145^ Therefore, scientists explored different reactive groups that are stable in vivo.

**Figure 15:**
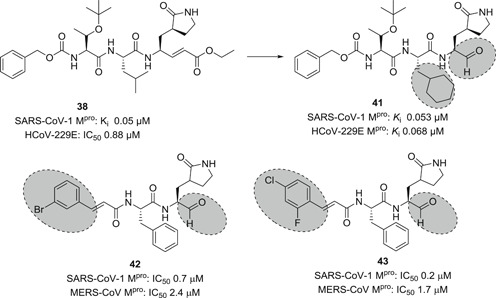
SARS‐CoV‐1 and MERS‐CoV M^pro^ inhibitors with peptide aldehyde functionality. M^pro^, main protease; SARS‐CoV, severe acute respiratory syndrome coronavirus

Based on the highly potent 1,4‐Michael‐acceptor‐based inhibitor **38**, which they had previously developed (see Figure 15), Yang et al.^134^ designed a peptide with a new efficient cysteine‐reactive group, using an aldehyde moiety. In addition, the P2‐leucine and the Michael groups of **38** were modified by a cyclohexyl unit and aldehyde group respectively to improve cellular activity. Indeed, the resulting peptide‐aldehyde **41** (Figure 15) showed remarkable activity against SARS‐CoV‐1 and HCoV‐229E M^pro^.^134^ It displayed promising antiviral activities decreasing viral load by 4.7 log (at 5 µM) for SARS‐CoV‐1 and 5.2 log (at 1.2 µM) for HCoV‐229E. This compound was stable in rat, mouse, and human plasma (even after 120 min, more than 70% of it remained in respective cells).

Kumar et al.^146^ reported another series of peptide‐aldehyde inhibitors with reduced molecular weight. Selected examples (**42**, **43**) are depicted in Figure 15. They were potent, cell‐membrane permeable, dual M^pro^ inhibitors of SARS‐CoV‐1 and MERS‐CoV, without cytotoxicity (CC_50_ > 100 µM). Compound **43**, in particular, revealed highly potent activity against SARS‐CoV‐1 M^pro^ (IC_50_, 0.2 µM) and MERS‐CoV M^pro^ (IC_50_, 1.7 µM). It displayed antiviral activity (EC_50_, 0.06 µM) lowering the viral load and the secretion of virus particles in MERS‐CoV‐infected cells. Also, it displayed broad‐spectrum antiviral activity against other human α‐ and β‐CoVs.

Akaji et al. discovered a series of SARS‐CoV‐1 M^pro^ inhibitors derived from its natural peptide substrate. Initially, they designed a pentapeptide (Ac‐Ser‐Val‐Leu‐N(CH_3_)_2_Gln‐CHO, **44**) with M^pro^ inhibitory activity of 37 µM.^147^ SAR studies of **44** led to inhibitor containing P1‐imidazole with improved potency (**45**; IC_50_, 5.7 µM). Further systematic structural modifications, primarily concentrating on P1‐, P2‐, and P4‐moieties, driven by X‐ray structure‐based analyses of the M^pro^‐inhibitor complex, led to the identification of inhibitor **46** with remarkable inhibitory activity (IC_50_, 98 nM). The crystal structure of M^pro^ with **46** revealed significant binding interactions in the active site. The P1‐imidazole nitrogen atom created a hydrogen bond with the histidine residue's imidazole nitrogen, and the P2‐cyclohexyl moiety fitted well into the S2‐subsite. This compound was characterized as a competitive inhibitor without covalent bond formation.

**Figure 16:**
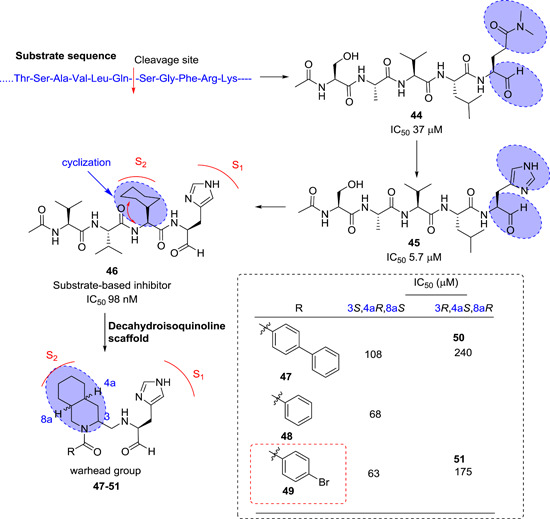
Peptide inhibitors containing cyclohexyl and decahydroisoquinoline groups [Color figure can be viewed at wileyonlinelibrary.com]

The same research group disclosed a novel series of peptide inhibitors containing a decahydroisoquinoline moiety in place of P2‐cyclohexyl of **46** to reduce the peptidic nature of the inhibitors. A few examples (**47**–**51**) are shown in Figure 16. Among them, **49** was moderately more active against SARS‐CoV M^pro^ when compared to **46**.^148^ The X‐ray structure of M^pro^ in complex with **49** revealed that the P2‐decahydroisoquinoline moiety was fittingly placed in the S2‐subsite, while the P1‐imidazole moiety occupied the S1‐subsite. With these key residues located appropriately in their respective pockets, the terminal functional group fits tightly into the active site.

This group further extended their study to find inhibitors that interact with S2 to S4 subsites. Taking **49** as a lead, they designed a new compound, by combining a nonprime substituent at the decahydroisoquinoline moiety, as shown in example **52**.^149^ The resulting **52** showed more than twofold increased M^pro^ inhibitory activity compared to **49**. This indicates that the additional interactions at S2–S4 sites enhance inhibitory activity.

Rather recently, the same research group explored the ability of octahydroisochromene to interact with the hydrophobic S2 pocket as an innovative P2‐moiety.^150^ To identify the best specific configuration, all possible diastereomers were evaluated. It was found that the molecule with (1*S*,3*S*)‐octahydroisochromene **53**–**56** could secure the optimal position of the P1‐imidazole as well as the aldehyde functional group at the active site. Additionally, the *N*‐butyl side chain attached to the 1‐position of the fused ring system was recognized to be important for establishing hydrophobic interactions.

**Figure 17:**
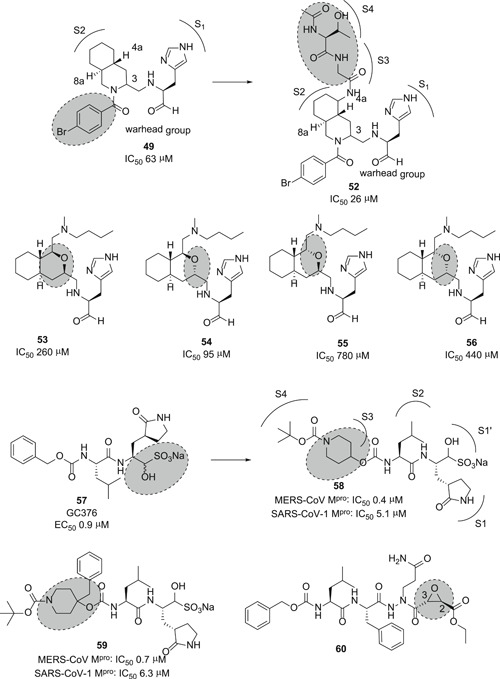
Inhibitors with aldehyde, aldehyde bisulfite adduct, and epoxide warhead group

In 2018, Groutas et al.^151^ disclosed a novel class of dual MERS‐CoV and SARS‐CoV‐1 M^pro^ inhibitors that contain a P3‐piperidine moiety (**58**–**59**; Figure 17). These inhibitors were derived from the dipeptidic‐aldehyde bisulfite adduct **57** (GC376), which was clinically studied as a protease inhibitor for its efficacy against CoVs such as the feline infectious peritonitis virus (FIPV). Compounds **58** and **59** showed potent antiviral activity toward MERS‐CoV in cell‐based bioassays (EC_50_, 0.5 µM for **58** and 0.8 µM for **59**). SAR studies revealed that the piperidine moiety engaged in favorable hydrophobic interactions at the S3 and S4 pockets of the protease.

The X‐ray crystal structures of MERS‐CoV 3CL^pro^ in complex with inhibitor **59** showed that the piperidine ring is likely projecting toward the S4 subsite. Additionally, **59** was engaged in backbone H‐bonds with Gln192, Gln167, and Glu169.

Azapeptide epoxides (APEs) are another class of SARS‐CoV‐1 M^pro^ inhibitors, although they were originally developed for clan CD cysteine peptidases.^152^, ^153^ The epoxide *S,S*‐diastereomer **60** (*K*_inact_/*K*_i_, 1900 (±400) M^−1^·s^−1^; Figure 17) exhibited the best inhibitory activity against SARS‐CoV M^pro^.^154^ The X‐ray structure of M^pro^ in complex with **60** confirmed the formation of a covalent bond between the cysteine‐S atom and the epoxide C‐3. It is worth noting that the *S,S*‐configured epoxide is required for the activity.

**Figure 18:**
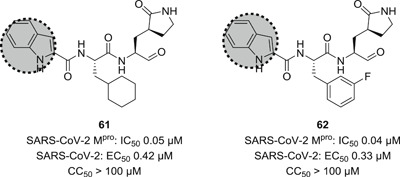
Peptidomimetic SARS‐CoV‐2 M^pro^ inhibitors with P3‐indole moiety. M^pro^, main protease; SARS‐CoV, severe acute respiratory syndrome coronavirus

Very recently, Dai et al. designed and synthesized two novel peptidomimetic SARS‐CoV‐2 M^pro^ inhibitors **61** and **62** (Figure 18) which exhibited extremely high inhibitory activity on purified M^pro^ with IC_50_ values of 50 and 40 nM, respectively. Furthermore, the group observed high antiviral activity of both compounds in cell‐based assays (**61**: EC_50_, 0.42 µM; **62**: EC_50_, 0.33 µM). X‐ray structures were determined for both derivatives in complex with SARS‐CoV‐2 M^pro^ at 1.5 Å, providing detailed information about the binding pockets. Similar to related molecules that employ the aldehyde moiety as a warhead, a covalent bond with the active‐site Cys145 was demonstrated for both structures. Cytotoxicity assays revealed CC_50_ values greater than 100 µM.^155^

#### 3.1.5 Ketoamide inhibitors

**Figure 19:**
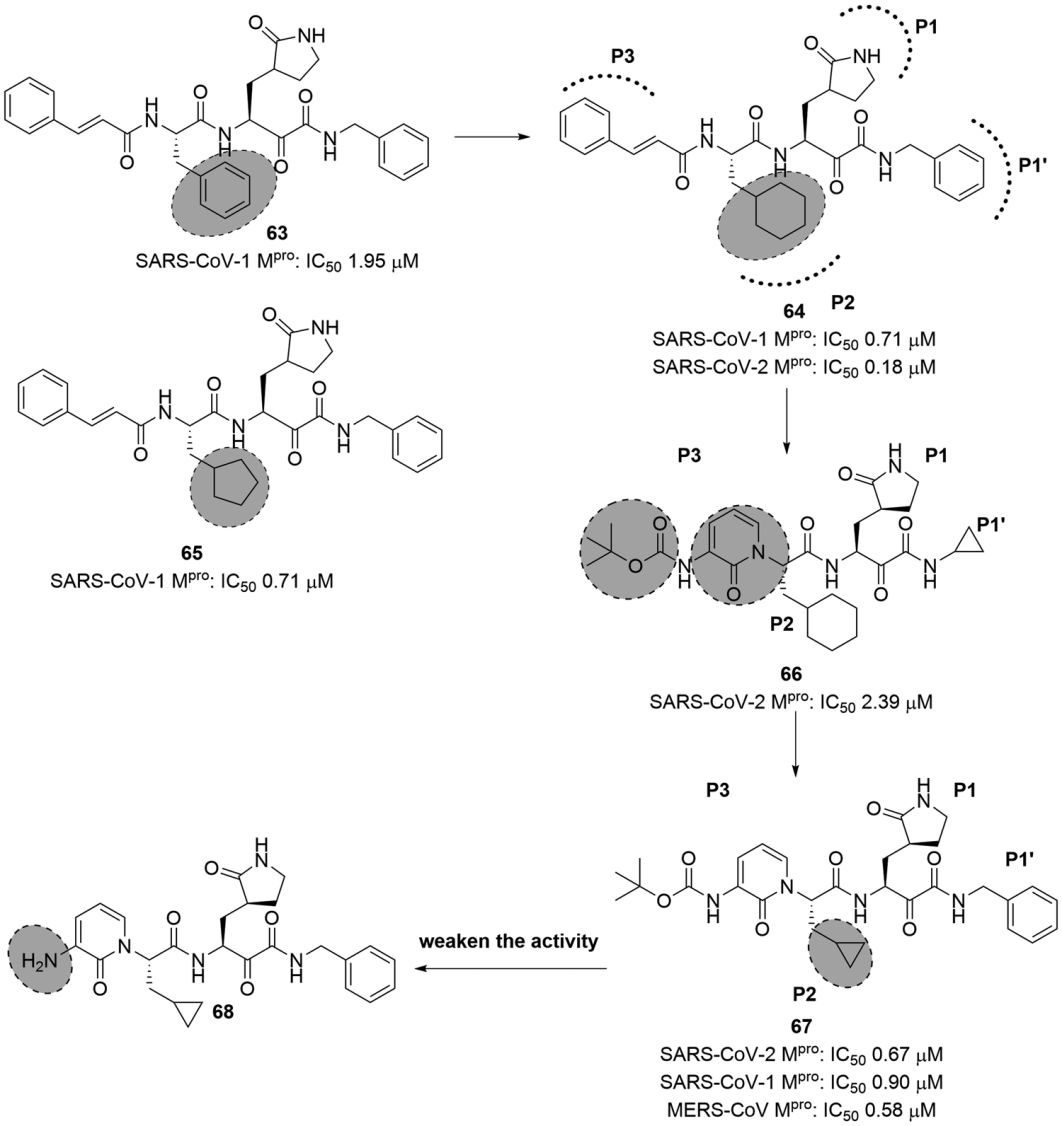
Ketoamide inhibitors targeting SARS‐CoV‐2 M^pro^. M^pro^, main protease; SARS‐CoV, severe acute respiratory syndrome coronavirus

Liu et al. reported dipeptidic α‐ketoamides as broad‐spectrum antiviral agents against the main proteases of human α and β‐CoVs as well as the 3C protease of enterovirus. The α‐ketoamide warhead group was promising, as it provides two hydrogen bond acceptors—one from the keto and one from the amide oxygen—whereas other warhead groups, such as Michael acceptor esters and aldehydes, provide only one hydrogen bond acceptor. Compound **63** was identified as SARS‐CoV M^pro^ inhibitor with an IC_50_ value of 1.95 µM (Figure 19).^156^

Taking **63** as a lead, aided by its X‐ray structure in complex with SARS‐CoV‐1, HCoV‐NL63, and coxsackievirus M^pros^, systematic structural modifications were investigated, focusing on the P2‐moiety. As a result, the replacement of P2‐phenyl with P2‐cyclohexyl (**64**) was found to be the best substitution, while P2‐cyclopentyl (**65**) showed similar potency against the enzyme SARS‐CoV‐1 M^pro^. In Huh7 cells, **64** also showed strong antiviral activity with an EC_50_ of 400 pM, but in Vero cells the antiviral activity of **64** was drastically reduced to 5 µM. This compound also exhibited antiviral activity against a range of enteroviruses in various cell lines.

**Figure 20:**
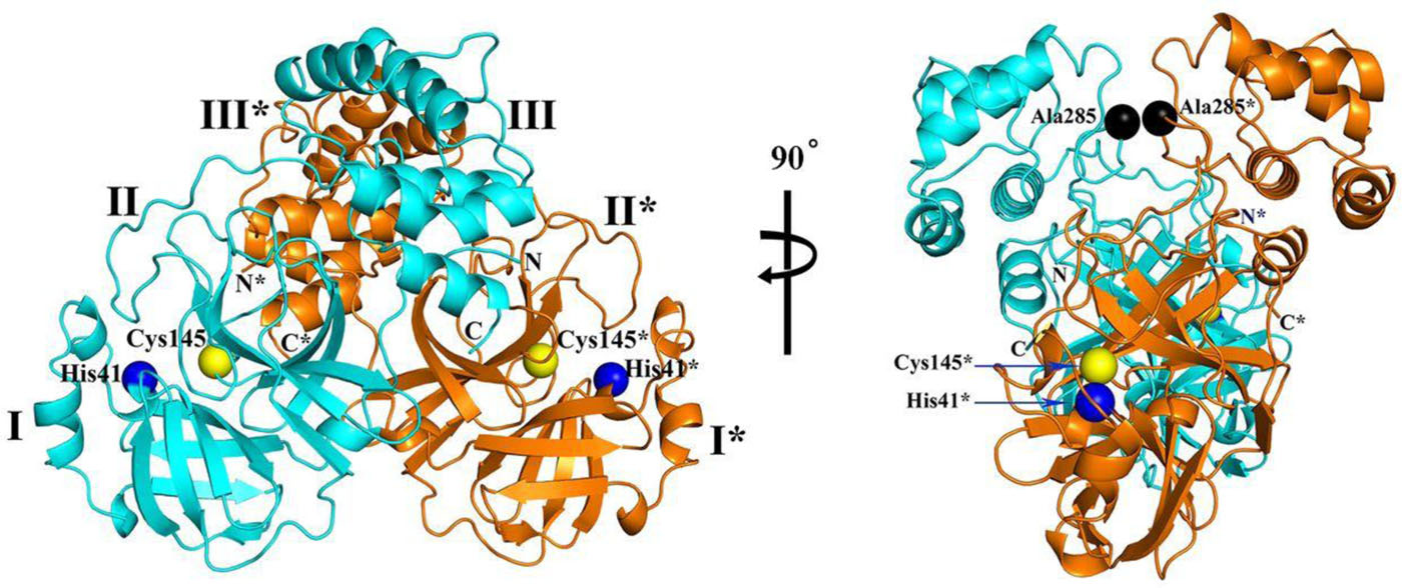
Crystal structure of SARS‐CoV‐2 M^pro^.^157^ M^pro^, main protease; SARS‐CoV, severe acute respiratory syndrome coronavirus [Color figure can be viewed at wileyonlinelibrary.com]

Due to the high similarity between SARS‐CoV‐1 M^pro^ and SARS‐CoV‐2 M^pro^ authors speculated that **64** was likely to inhibit the new virus as well. Zhang et al. recently reported this molecule as a SARS‐CoV‐2 M^pro^ inhibitor with an IC_50_ value of 0.18 µM. They first resolved the unliganded crystal structure of SARS‐CoV‐2 M^pro^ (Figure 20),^157^ which is largely identical to that of SARS‐CoV‐1 M^pro^ with a 96% sequence identity. Compound **64** was docked to SARS‐CoV‐2 M^pro^, and a series of structural modifications were performed to improve its pharmacokinetic properties. Specifically, masking the P2‐P3 amide bond with the pyridone ring could improve plasma half‐life; and exchanging the lipophilic cinnamoyl residue for the less lipophilic Boc group, could increase plasma solubility and reduce its binding to plasma proteins.

Indeed, the resulting **66** had a ~3‐fold improved plasma half‐life in mice when compared to the lead **65** (from 18 min to 1 h). The in vitro kinetic plasma solubility has been increased by a factor of ~19 (from 6 µM for the lead to 112 µM for best derivative), and the thermodynamic solubility by a factor of ~13 (from 41 to 530 µM). Compound **66** also showed reduced binding to mouse plasma protein. However, compared to the lead (IC_50_, 0.18 µM), the structural modifications caused a reduction of activity against SARS‐CoV‐2 M^pro^ (IC_50_, 2.39 µM) and enteroviral 3 C proteases. Nevertheless, the introduction of a cyclopropyl group as in **67** instead of P2‐cyclohexyl enhanced the antiviral activity against β‐coronaviruses.

Compound **67** (Figure 19) inhibited purified SARS‐CoV‐2 M^pro^ with an IC_50_ of 0.67 µM. It also inhibited SARS‐CoV‐1 M^pro^ (IC_50_, 0.90 µM) and MERS‐CoV M^pro^ (IC_50_, 0.58 µM) with similar potency. It was effective against SARS‐CoV‐1 replication with an EC_50_ value of 1.75 µM. In SARS‐CoV‐2 infected human Calu3 cells, it inhibited the viral replication with an EC_50_ of 4–5 µM, when in fact the Boc‐unprotected **68** was inactive, suggesting a bulky hydrophobic group is necessary for cellular membrane penetration. On the other hand, increasing hydrophobicity of molecules should be pondered carefully, as it can increase plasma protein binding as it was described for **64**. The pharmacokinetic properties of **67** revealed striking lung tropism and was suitable for inhalation in mice without any perceived adverse effects.

**Figure 21:**
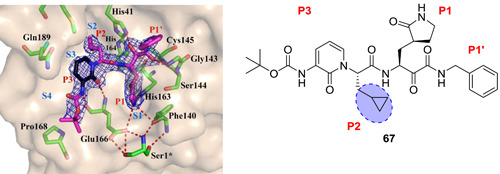
Crystal structure of **67** with SARS‐CoV‐2 M^pro^. M^pro^, main protease; SARS‐CoV, severe acute respiratory syndrome coronavirus [Color figure can be viewed at wileyonlinelibrary.com]

Compound **67** was cocrystallized with the enzyme in two different forms at 1.95 and 2.20 Å (Figure 21). The key feature observed from this crystal structure was that the inhibitor binds to the shallow substrate‐binding site at the surface of each protomer, between domains I and II. The thioketal that resulted from the nucleophilic Cys145 attacking the inhibitor, is stabilized by a H‐bond from His41, whereas the amide oxygen of **67** accepts a H‐bond from the main‐chain amides of Gly143, Cys145, and in part, Ser144 that make up the cysteine protease's canonical oxyanion hole.^157^ The P1 lactam moiety is deeply embedded in the S1 pocket where the lactam nitrogen donates a three‐center H‐bond to the main chain oxygen of the Phe140 and the carboxylate of Glu166. The carbonyl oxygen forms a H‐bond to His163. The P2‐cyclopropyl moiety fits into the S2 subsite. The P3‐P2 pyridone moiety occupies the space normally filled by the substrate's main chain. The Boc group is not situated in the canonical S4 site, rather it is located near Pro168, which explains why the removal of the Boc group as in **68** weakened the inhibitory activity.

#### 3.1.6 Inhibitors with electrophilic ketone

**Figure 22:**
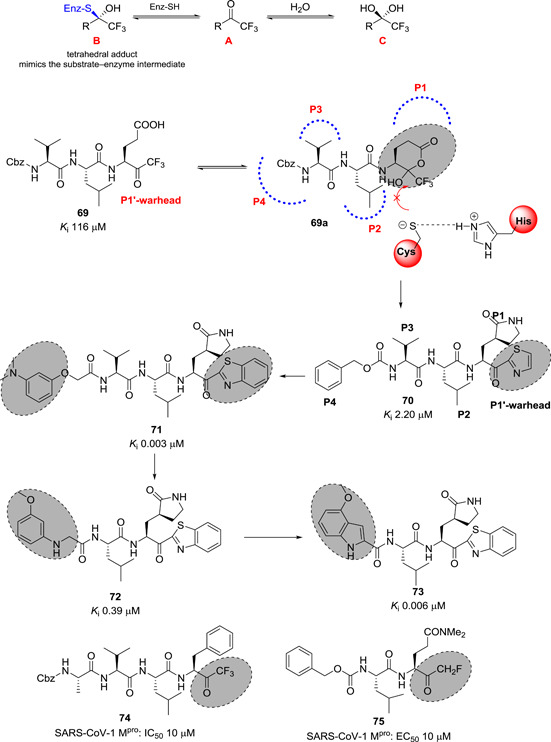
Peptide inhibitors containing electrophilic ketone warheads [Color figure can be viewed at wileyonlinelibrary.com]

It was envisioned that a fluorinated ketone moiety could be utilized as a warhead for targeting proteases, because it forms a thermodynamically stable hemiketal or hemithioketal after nucleophilic attack by Ser‐OH or Cys‐SH residues, which are present in the active sites of serine or cysteine proteases, respectively (see Figure 22).

Initially, Hayashi et al. reported a series of natural‐substrate‐derived peptide inhibitors containing a trifluoromethyl ketone warhead targeting SARS‐CoV‐1 M^pro^. Compound **69** (Figure 22) was the best of the series with a *K*_i_ value of 116 µM against SARS‐CoV‐1 M^pro^.^158^ It was sequentially modified mainly focusing on the warhead moiety since the formation of a cyclic structure prevented the nucleophilic attack by cysteine at the active site. This study led to the discovery of **70** containing a P1‐lactam and P1'‐thiazole moiety with a >50‐fold increase in inhibitory activity compared to **69**.^159^ Docking studies of **70** to M^pro^ highlighted key H‐bond interactions with backbone amino acid residues Cys143, Ser144, and Cys145. The nitrogen atom of the thiazole warhead moiety also engaged in H‐bond interactions, and the P1‐lactam nicely fitted into the S1‐pocket.

**Figure 23:**
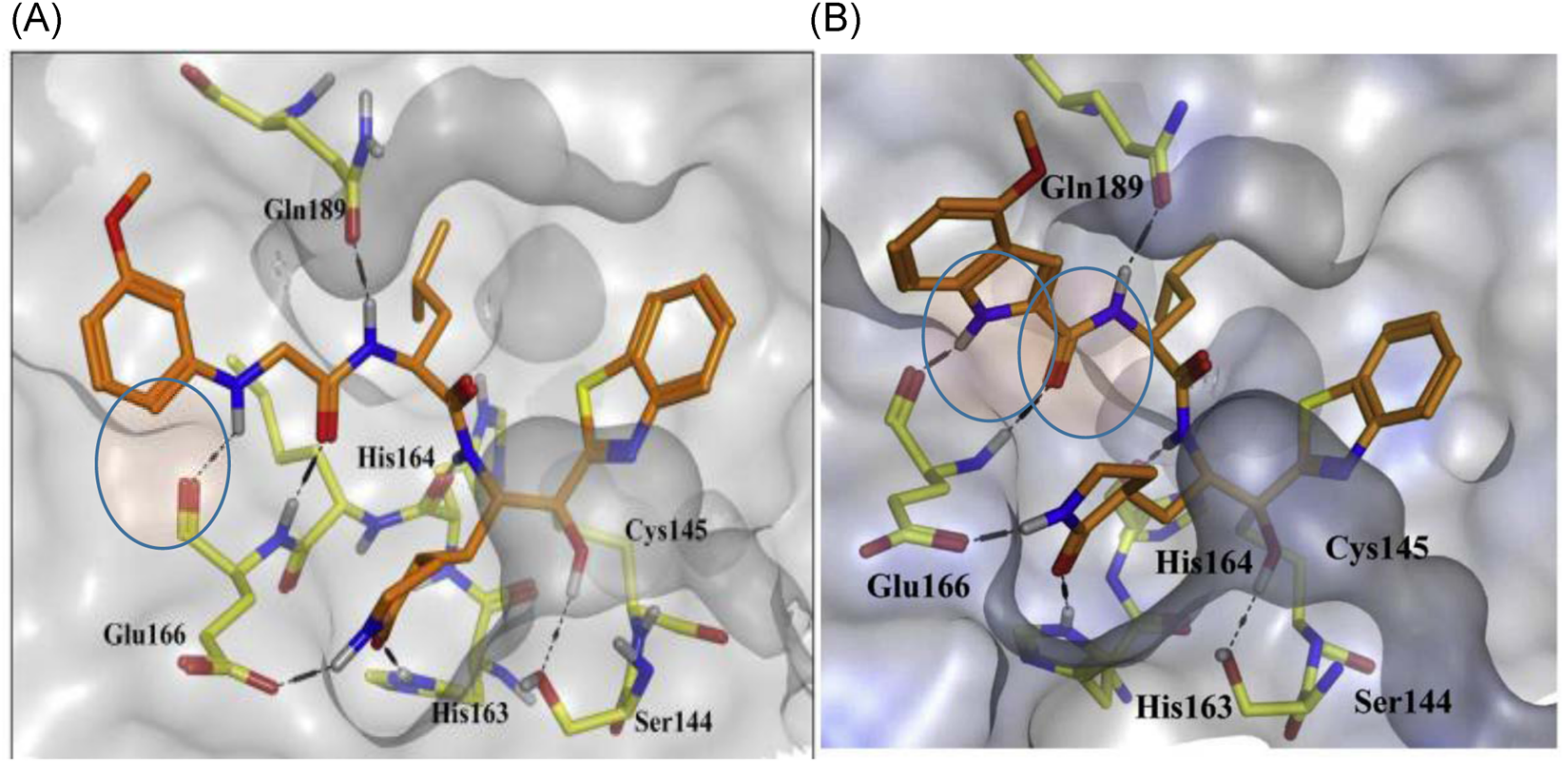
(A) Docking poses of **72** and (B) **73** with SARS‐CoV‐1 M^pro^. M^pro^, main protease; SARS‐CoV, severe acute respiratory syndrome coronavirus [Color figure can be viewed at wileyonlinelibrary.com]

Continued computer‐assisted structural design led to a tripeptide containing benzothiazole as a warhead group and an *m*‐*N,N*‐dimethylaminophenyl group as P4‐moiety (**71**).^160^ This compound was extremely potent in inhibiting M^pro^ of SARS‐CoV‐1 with a *K*_i_ value of 3.1 nM. Docking studies of **71** confirmed that the benzothiazole group was tightly bound to the active site. Consequently, the same research group disclosed a series of dipeptides with reduced molecular weight in an attempt to improve drug‐like properties. The P3‐valine in the tripeptide **71** was exchanged for a variety of functional groups.^161^ The study determined *N*‐arylglycyl to be the optimal P3‐moiety. Compound **72** displayed the best inhibitory activity. Docking studies of **72** to the protease highlighted the amino hydrogen of the P3‐*N*‐phenyl glycyl forming a H‐bond with backbone Glu166 of M^pro^, in addition to the best P2‐leucine and P1'‐benzthiazole moieties (see Figure 23A). Further structural optimization at the P3‐*N‐*arylglycyl moiety found the indole‐2 carbonyl group to be one of the best P3‐moeities, thus reaching inhibitors with low nanomolar potency, for example **73** (*K*_i_, 0.006 µM) against SARS‐CoV‐1 M^pro^.^162^ Docking studies of compound **73** to the protease revealed that the indole amino hydrogen and the carbonyl group attached to the 2‐position formed H‐bond interactions with the backbone Glu166 (see Figure 23B). These interactions are of great importance, seeing as shifting the position of the carbonyl group from position 2 to 3, or replacing the indole with benzofuran drastically reduced inhibitory potency.

Zhao et al. reported a series of trifluoromethyl ketones. Among them, **74**, which has the same sequence as the peptide substrate from sites P1 to P4, exhibited moderate inhibitory activity with an IC_50_value of 10 µM. Inhibitor **74** also displayed time‐dependent inhibition (*K*_i_, 0.3 µM).^163^

Zhang et al. described a series of dipeptides containing difluoromethyl ketone as SARS‐CoV‐1 M^pro^ inhibitors. Compound **75** displayed the best inhibitory activity in infected Vero and Caco‐2 cell cultures with an IC_50_ value of 2.5 µM. It also exhibited little toxicity.^164^

A library of small peptide‐anilides was developed as anti‐SARS‐CoV‐1 M^pro^ agents (**77**–**80**; Figure 23). These inhibitors were basically designed from niclosamide (**76**) which was inactive at M^pro^ of SARS‐CoV‐1. Proper structural modifications led to the discovery of **77** (IC_50_, 0.06 µM). It behaved as a competitive, noncovalent inhibitor (*K*_i_, 0.03 µM). SAR investigations pointed out that the *N,N*‐dimethyl group on the phenyl ring, and electron‐withdrawing groups at the warhead phenyl are important. Structural modification of **77** resulted in compounds **78** *–* **80** displaying reduced potency.^165^

**Figure 24:**
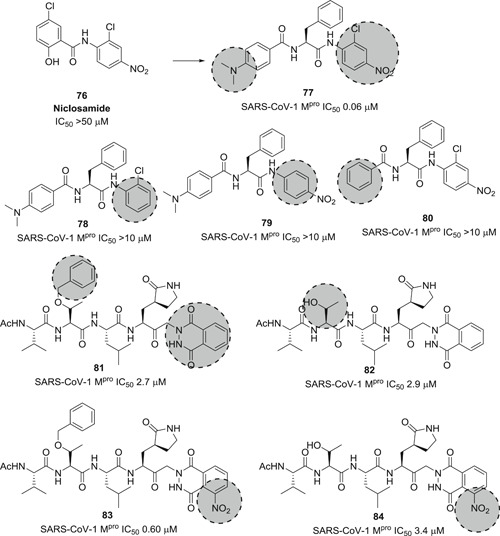
Small peptide anilides and ketoglutamide tripeptides as SARS‐CoV‐1 inhibitors. SARS‐CoV, severe acute respiratory syndrome coronavirus

A novel series of ketoglutamide tripeptides bearing a phthalhydrazido warhead group were identified as reversible SARS‐CoV‐1 M^pro^ inhibitors (**81**–**84**; Figure 24).^166^ Among them, compound **83** showed the best inhibition (IC_50_, 0.6 µM). SAR studies revealed the presence of β and β'‐amino functionality adjacent to the keto and the intramolecular hydrogen bond to the carbonyl group made the keto center more electrophilic and inclined to build a hemithioacetal with Cys‐SH at the active site. Additionally, the hydrophobic P3‐benzyloxy moiety, the P1‐lactam, and the nitro group significantly contributed to the activity increment.

**Figure 25:**
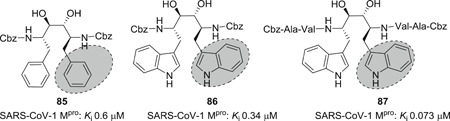
SARS‐CoV‐1 M^pro^ inhibitors derived from HIV proteases inhibitors. HIV, human immunodeficiency virus; M^pro^, main protease; SARS‐CoV, severe acute respiratory syndrome coronavirus

Wang et al.^167^ described the development of selective and reversible SARS‐CoV‐1 M^pro^ inhibitors derived from HIV proteases inhibitors (Figure 25). The compound **85** as a SARS‐CoV‐1 M^pro^ lead inhibitor was continuously modified to obtain **86** and **87**. These derivatives were highly selective toward SARS‐CoV‐1 M^pro^ versus HIV protease. Docking studies of **87** to M^pro^ demonstrated that both indole amino hydrogens establish H‐bond networks with side chain His142 and His41.

#### 3.1.7 Small molecule inhibitors of M^pro^

**Figure 26:**
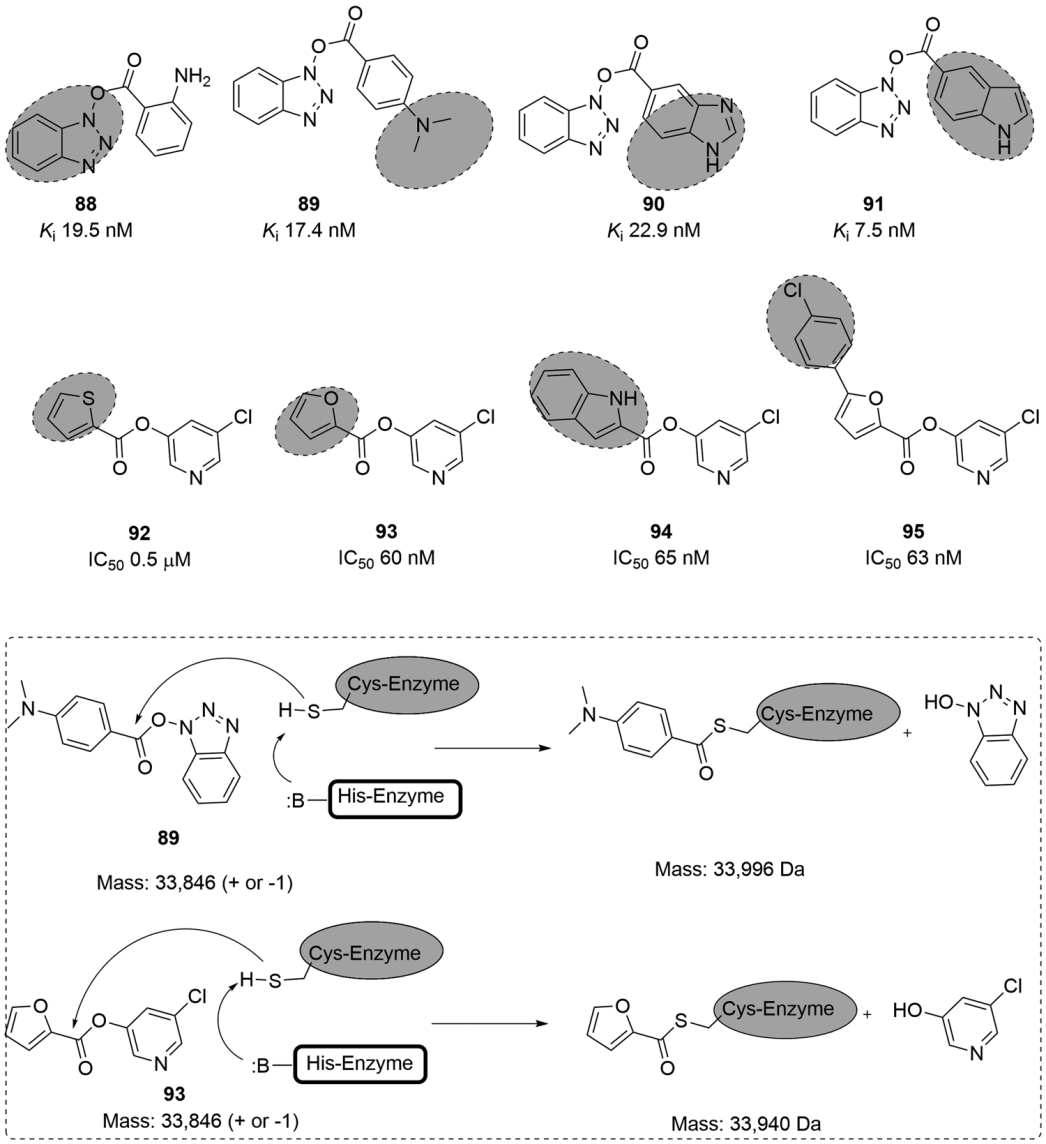
Active esters as SARS‐CoV‐1 M^pro^ inhibitors. M^pro^, main protease; SARS‐CoV, severe acute respiratory syndrome coronavirus

Benzotriazole esters (**88**–**91**; Figure 26) were discovered as novel nonpeptidic irreversible inhibitors of SARS‐CoV‐1 M^pro^.^168^ Among them, **91** exhibited the best enzymatic inhibitory activity, but no antiviral activity in cell‐based assays. The covalent binding mode of **91** was confirmed by electrospray ionization mass spectrometry (ESI‐MS) analyses.

With a slight structural modification from benzotriazole ester, Zhang et al. reported a series of active halopyridyl esters containing thiophene, furan, and indole moieties (**92**–**95**; Figure 26). Among them, **93** displayed the highest enzymatic inhibitory activity at SARS‐CoV‐1 M^pro^.^169^ However, no antiviral activity for this compound was communicated. The irreversible binding mode of **93** was confirmed by ESI‐MS analysis.^170^, ^171^

**Figure 27:**
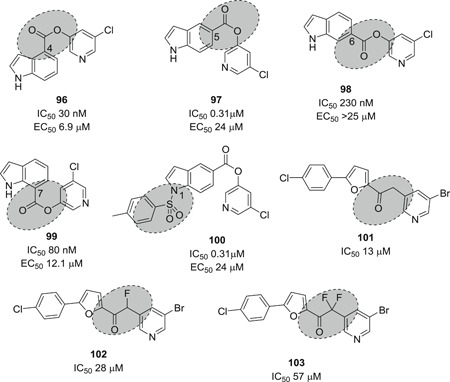
SAR of halopyridinyl indole carboxylates as SARS‐CoV‐1 M^pro^ inhibitors. M^pro^, main protease; SARS‐CoV, severe acute respiratory syndrome coronavirus

Ghosh et al.^172^ studied the SARs of halopyridinyl indole carboxylates and identified a series of analogs (**96**–**101**; Figure 27) as SARS‐CoV‐1 M^pro^ inhibitors in the nanomolar potency range. The best derivative (**100**) had high enzymatic inhibitory potency (IC_50_, 0.030 µM) and antiviral activity (EC_50_, 6.9 µM). Compound **97** was also observed to inhibit the MERS‐CoV M^pro^ both in enzymatic and cell‐based (EC_50_, 12.5 µM) bioassays.^173^ This molecule covalently modified M^pro^, which was confirmed by MALDI‐TOF studies.

**Figure 28:**
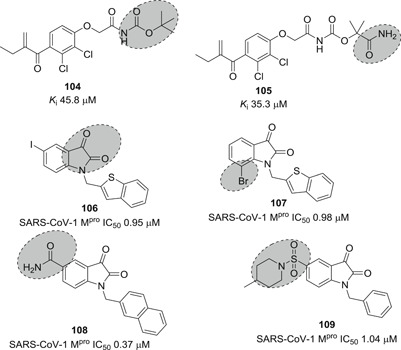
Etacrynic acid and isatin derivatives as SARS‐CoV‐1 M^pro^ inhibitors. M^pro^, main protease; SARS‐CoV, severe acute respiratory syndrome coronavirus

5‐Halopyridinyl esters are troublesome drug candidates because of their potential for rapid hydrolysis by various esterases and other enzymes in mammalian cells. They can potentially also react nonspecifically with other thiols and nucleophiles, a recipe for cytotoxicity. To bypass this problem by developing stable noncovalent inhibitors, Zhang et al.^174^ reported a group of methylene ketones and analogous mono‐ and di‐fluorinated methylene ketones based on pyridinyl esters (**102** and **103**; Figure 28) as SARS‐CoV‐1 M^pro^ inhibitors. Enzymatic investigations and ESI‐MS experiments illustrate that those inhibitors bind to their target in a noncovalent, reversible manner.

An HPLC‐based screening of electrophilic compounds identified the etacrynic acid‐derived amide **106** and ester **107** as SARS‐CoV‐1 M^pro^ inhibitors with moderate potency.^175^ Etacrynic carboxamide (**105**; *K*_i_, 35.3 µM) bound more strongly to SARS‐CoV‐1 M^pro^ than to papain protease, while etacrynic acid ester **104** was more active at papain protease (*K*_i_, 3.2 µM) than at SARS‐CoV‐1 M^pro^ (*K*_i_, 45.8 µM; Figure 28). SAR studies suggested that chloro substituents were necessary for protease inhibition. Docking studies of **105** to M^pro^ revealed that it forms hydrogen bonds with Gln189, Glu166, Thr190, and Gln192 with its terminal amino group. The Michael system carbonyl group interacts with Gly143, and the reactive double bond remained next to the Cys145 sulfur.

Previously, isatin (2,3‐dioxoindole) derivatives were observed to inhibit rhinovirus 3C protease.^176^ Due to the structural similarity between the rhinovirus 3C protease and SARS‐CoV‐1 M^pro^, these derivatives were tested against SARS‐CoV‐1 M^pro^. Among them, **106** (IC_50_, 0.95 µM) and **107** (IC_50_, 0.98 µM) exhibited the best SARS‐CoV‐1 M^pro^ inhibitory activity in the low micromolar range.^176^ SAR studies suggested that the inhibition efficiency was mainly reliant on hydrophobic and electronic properties of the isatin core substitution pattern. Docking studies revealed that the molecules fit well in the active site of the protease. Both carbonyl groups of the isatin core engaged in H‐bonds with NH of Gly143, Ser144, Cys145, and His41. Compounds **106** and **107** ^176^ were more selective for SARS‐CoV‐1 M^pro^ than other proteases like papain (**106**, 103 µM; **107**, 87.24 µM), chymotrypsin (**106**, ~1 mM; **107**, 10.4 µM), and trypsin (**106**, 362 µM; **107**, 243 µM; Figure 28).

Zhou et al. extended the SAR studies for further activity improvement. Compound **108** bearing carboxamide showed the best SARS‐CoV‐1 M^pro^ inhibitory activity. However, this derivative did not bind covalently to the Cys145 residue of the active site.^177^ Further structural investigations at the carboxamide of **108** with a variety of substituted sulfonamides did not improve the activity. Compound **109** was the best one of that series (Figure 28).^178^

**Figure 29:**
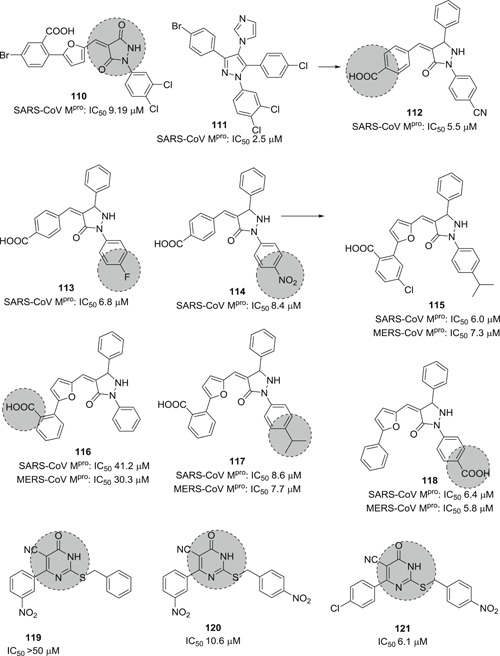
Pyrazoles and pyrimidines as SARS‐CoV‐1 M^pro^ inhibitors. M^pro^, main protease; SARS‐CoV, severe acute respiratory syndrome coronavirus

The modification of **110**, identified by high‐throughput screening (HTS; Figure 29), led to pyrazolone and pyrazole derivatives **111** and **112** as SARS‐CoV‐1 M^pro^ inhibitors.^179^, ^180^ Taking these as leads, Ramajeyam et al.^181^ reported compounds **112**–**114** to be the best‐performing inhibitors of the series(IC_50_ 5.5, 6.8, 8.4 µM, respectively). They also observed moderate inhibitory activity against CVB3 3C^pro^. Structure‐functionality analyses illustrated that the benzylidene ring next to pyrazolone C4 in addition to electron‐withdrawing groups, favors inhibitory activity. Molecular modeling studies of **112** predicted that for its inhibitory function, the *N1*‐phenyl residue in the M^pro^ S1 site as well as the carboxyl benzylidene moiety in the S3 pocket are important.

Kumar et al. described furan‐inserted pyrazolone derivatives as dual SARS‐CoV‐1 M^pro^ and MERS‐CoV M^pro^ inhibitors (**115**–**118**; Figure 29).^182^ Compounds **115**, **117**, and **118** exhibited the best dual inhibitory activities. Compounds **115** and **116** also displayed inhibitory activity against H5N1 neuraminidase (IC_50_ 2.8, 2.9 µM, respectively).^183^ Ramajeyam et al. also disclosed a range of pyrimidine derivatives as SARS‐CoV‐1 M^pro^ inhibitors (**119**–**121**). Compound **121** showed high inhibitory potency with an IC_50_ value 6.1 µM.^181^

**Figure 30:**
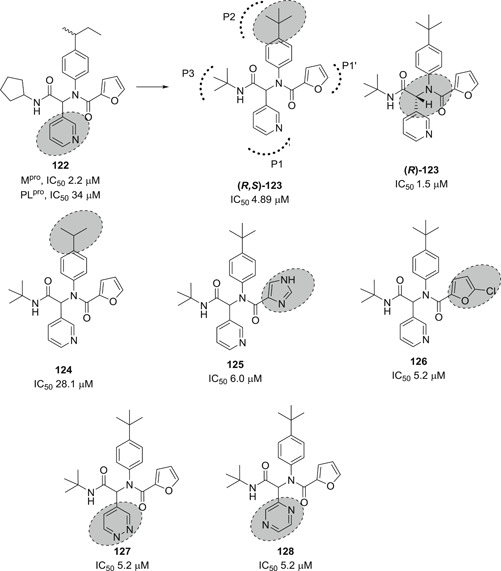
Simple dipeptide derivatives as SARS‐CoV‐1 M^pro^ inhibitors. M^pro^, main protease; SARS‐CoV, severe acute respiratory syndrome coronavirus

HTS of NIH molecular libraries (~293 000 substances) yielded the dipeptide **122** containing 3‐pyridyl as hit compound against SARS‐CoV‐1 M^pro^ with an IC_50_ value of 2.2 µM (Figure 30). Preliminary SAR studies identified **123** and **124** as the most promising inhibitors of the series.^184^, ^185^

**Figure 31:**
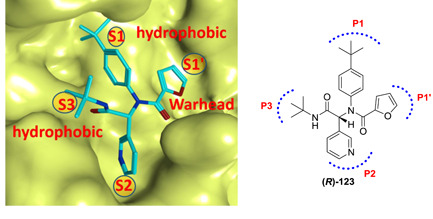
The X‐ray crystal structure of **123** bound to the binding pocket of SARS‐CoV‐1 M^pro^ (PDB ID: 3V3M). Pockets S1'–S3 are highlighted. M^pro^, main protease; SARS‐CoV, severe acute respiratory syndrome coronavirus [Color figure can be viewed at wileyonlinelibrary.com]

The X‐ray crystal structure of **123** attached to SARS‐CoV‐1 M^pro^ highlighted the compound's identical orientation in the pocket to that of established covalent peptidomimetic inhibitors (Figure 31). The compound with an *R*‐configuration occupied the S3‐S1' subsites of SARS‐CoV‐1 M^pro^. Indeed, only (*R*)‐**123** was able to inhibit the M^pro^ enzyme with an IC_50_ value of 1.5 µM, while the (*S*)‐enantiomer was inactive. (*R*)‐**123** inhibited SARS‐CoV‐1 M^pro^ in a competitive manner (*K*_i_, 1.6 µM) with a noncovalent mode of inhibition. (*R*)‐**123** also showed antiviral activity (12.9 µM) in mock infected and SARS‐CoV‐1 infected Vero E6 cells.

To enhance the inhibitory activity, SAR study efforts around P1' of **123** provided compounds containing imidazole (**125**) and 5‐chlorofuran (**126**) with equipotent activity to lead **123** (Figure 30). Next, the exploration of P1 3‐pyridyl unit of **123** revealed pyridazine (**127**) and pyrazine (**128**) which were only tolerated, albeit without any improvement.

**Figure 32:**
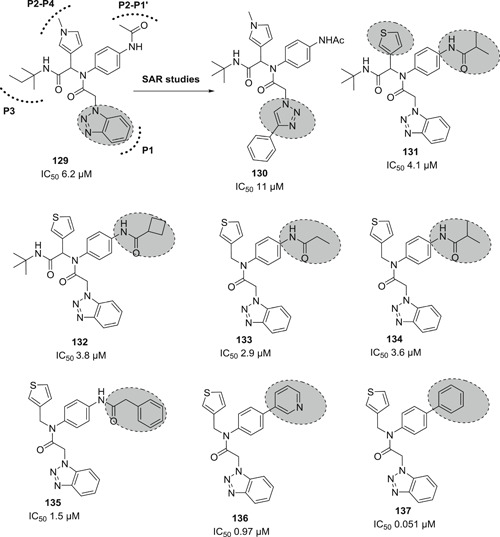
SARS‐CoV‐1 M^pro^ inhibitors containing the benzotriazole scaffold. M^pro^, main protease; SARS‐CoV, severe acute respiratory syndrome coronavirus

The same group of researchers discovered potent, noncovalent SARS‐CoV‐1 M^pro^ blockers based on a benzotriazole scaffold in an MLPCN screening,^186^ resulting in hit compound **129** (Figure 32) with a SARS‐CoV‐1 M^pro^ IC_50_ value of 6.2 µM.

SAR studies focusing on the benzotriazole moiety of **129** were performed to improve activity. The replacement of this group with 4‐phenyl‐1,2,3‐triazole (as in **130**) was somewhat tolerated (IC_50_ of 11 µM; Figure 32). Further modifications to the acetamide (P2‐P1' region) resulted in molecules bearing a thiophene ring on one side and a branched *i*‐propyl amide (**131**) or cyclobutylamide (**132**) on the other—reaching IC_50_ values below 5 µM.

To cut overall molecular weight of the inhibitors, P3‐truncation was performed, which led to potent derivatives (**133**–**137**; Figure 32). Compound **137** displayed extremely high inhibition (IC_50_, 51 nM).

**Figure 33:**
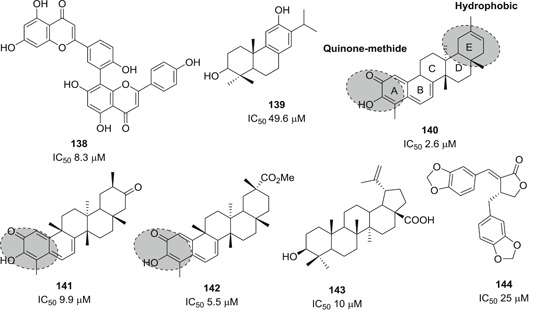
Flavone and terpenoid derivatives with inhibitory activity against SARS‐CoV‐1 3CL^pro^. 3CL^pro^, 3C‐like protease; SARS‐CoV, severe acute respiratory syndrome coronavirus

SARS‐CoV‐1 M^pro^ inhibitors were also discovered from medicinal plants. In 2011, Ryu et al.^187^ disclosed a range of inhibitors obtained from *Torreya nucifera* leaves. Of all the isolated chemicals, the biflavone, amentoflavone (**138**; Figure 33), was identified as a potent noncompetitive inhibitor with an IC_50_ of 8.3 µM. Docking studies of **138** identified the interactions of Val186 and Gln192 as major sites at the target.

They also isolated a series of terpenoids from *T. nucifera* as anti‐SARS‐CoV M^pro^ agents (Figure 33).^187^ Among them, ferruginol (**139**; IC_50_ 49.6 µM) was the most active compound. Additionally, they isolated quinone‐methide triterpenoids celastrol (**140**), pritimererin (**141**), and tingenone (**142**) from methanol extracts of *Tripterygium regelii* which exhibited fair inhibition activity (IC_50_ 2.6, 9.9, 5.5 µM, respectively). SAR studies indicated that for effective inhibition, the quinone‐methide group in ring A and the more lipophilic ring E were critical. All compounds were characterized as competitive inhibitors using kinetic analyses.

Wen et al.^188^ reported abietane‐type diterpenoids and lignoids with a powerful anti‐SARS‐CoV‐1 M^pro^ effect. Especially betulinic acid (**143**) and savinin (**144**) effectively inhibited SARS‐CoV‐1 M^pro^ (*K*_i_ 8.2 µM, 9.1 µM, respectively) (Figure 33). These inhibitors acted in a competitive manner.

**Figure 34:**
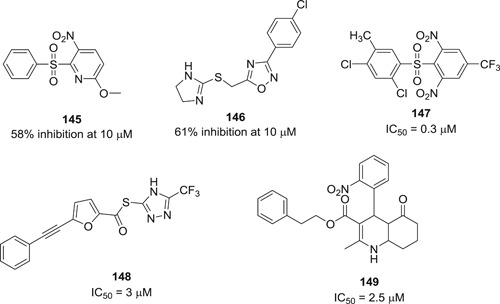
Structure of SARS‐CoV‐1 M^pro^ inhibitors **145**–**149**. M^pro^, main protease; SARS‐CoV, severe acute respiratory syndrome coronavirus

Lu et al. discovered two hit SARS‐CoV‐1 3CL^pro^ inhibitors, sulfone **145** and dihydroimidazole **146**, by structure‐based virtual screening of a compound library of 58 855 chemicals (Figure 34).^189^ The central structural elements of the hits, determined in docking experiments, were then used for additional analog searches.

Computational similarity screening discovered 21 analogs from these hits. Among them, the two best compounds **147** and **148** display IC_50_ values of 0.3 and 3 µM, respectively. A variety of SARS‐CoV‐1 M^pro^ inhibitors have been identified through virtual screening (VS) as an alternative to HTS. VS of 50 240 structurally diverse small molecules allowed to identify 104 molecules with anti‐SARS‐CoV‐1 activity. Compound **149** (Figure 34) demonstrated potent enzyme inhibition (IC_50_, 2.5 μM) and an EC_50_ of 7 μM in Vero cell‐based SARS‐CoV‐1 plaque reduction assays

**Figure 35:**
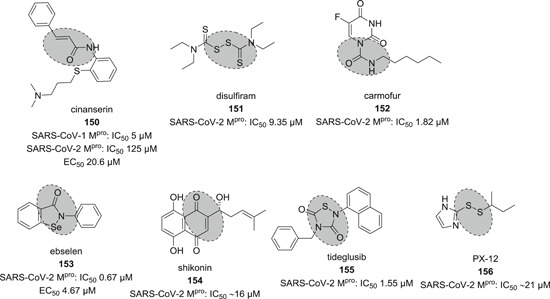
Covalent bond inhibitors of M^pro^. M^pro^, main protease

Virtual screening identified the serotonin antagonist cinanserin (**150**, Figure 35) as a potential inhibitor of M^pro^. It had previously shown activity against SARS‐CoV‐1 M^pro^ with an IC_50_ value of 5 µM.^190^ Subsequent tests revealed its anti‐SARS‐CoV‐2 activity (EC_50_, 20.6 µM) and an IC_50_ value of 125 µM (SARS‐CoV‐2 M^pro^).

Their HTS yielded seven primary hits including the approved drugs disulfiram (**151**) and carmofur (**152**), as well as ebselen (**153**), shikonin (**154**), tideglusib (**155**), and PX‐12 (**156**) (Figure 35).

Using MS/MS analysis, they deduced that ebselen (**153**) and **156** are irreversible inhibitors of M^pro^ by covalently attaching to Cys145 of the catalytic dyad. Molecular docking was used to illustrate how **151**, **154**, and **155** bind to M^pro^. Antiviral activity assays, using real‐time reverse transcription‐PCR, indicated that ebselen and inhibitor “N3” (**40**; Figure 12) had the strongest antiviral effects. Ebselen displayed an EC_50_ value of 4.67 µM, and “N3” showed an EC_50_ value of 16.77 µM in a plaque‐reduction assay. Ebselen's IC_50_ value for SARS‐CoV‐2 M^pro^ was reported at 0.67 µM. The activity data of remaining compounds is summarized in Figure 35.

Ebselen has been studied for an array of diseases and has a very low toxicity.^191^, ^192^, ^193^ Its safety has been demonstrated in clinical trials.^191^, ^192^, ^194^ It can therefore be considered a promising molecule for the treatment or prevention of CoV infections.

### 3.2 Coronavirus PL^pro^ inhibitors

Along with the M^pro^, papain‐like protease (PL^pro^) also cleaves polyproteins which is an important process for viral replication. PL^pro^ cleaves at the first three positions creating three nonstructural functional proteins (nsp1‐nsp3). In particular, nsp3 is central for the generation of the viral replication complex. The multifunctionality of PL^pro^ in deubiquitinating, de‐ISGylation (ISG: interferon‐stimulated gene),^195^, ^196^ and in the evasion of the innate immune response make PL^pro^ an attractive antiviral drug target.

PL^pro^ is a cysteine protease and its active site contains a catalytic triad composing of Cys112‐His273‐Asp287. Cys112 behaves as a nucleophile, and His273 is a general acid‐base. Asp287 helps His273 to align perfectly, thus promoting His to deprotonate Cys‐SH.

**Figure 36:**
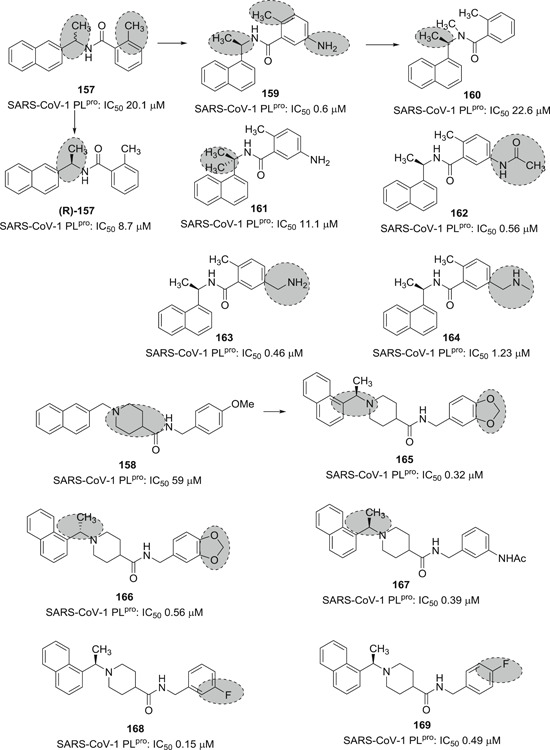
SARS‐CoV‐1 PL^pro^ inhibitors based on naphthalene scaffold. PL^pro^, papain‐like protease; SARS‐CoV, severe acute respiratory syndrome coronavirus

Ghosh et al.^197^ contributed significantly to the development of SARS‐CoV‐1 PL^pro^ inhibitors based on the naphthalene scaffold. Two lead compounds **157** and **158** (Figure 36) were identified by an HTS of a chemical library containing greater than 50 000 compounds. They both inhibit PL^pro^ of SARS‐CoV‐1 at a moderate potency (IC_50_ 20.1 and 59 µM, respectively). The (*R*)‐enantiomer of compound **157** was found to be a greater than twofold more potent inhibitor of PL^pro^ when compared with its racemic mixture (**157**). Subsequent SAR studies highlighted the 2‐naphthyl substitution as an important structural requirement rather than at the position 1 of the naphthyl ring in addition to the presence of *o*‐methyl and *m*‐amino groups, in the other phenyl ring. Compound **159** displayed the best inhibitory activity of PL^pro^ (IC_50_, 0.6 µM) and acts in a noncovalent reversible manner with a *K*_i_ value of 0.49 µM.^198^ Compound **159** also showed moderate antiviral activity in Vero cells with an EC_50_ value of 14.5 µM.

Compound **159** was further scrutinized by investigating the importance of the amide NH, and the effect of the substituent on the benzamide ring (**160**–**164**). Among them, compounds **163** and **164** exhibited the most potent enzymatic (**163**: IC_50_, 0.46 µM; **164**: IC_50_, 1.23 µM) and cell‐based antiviral (**163**: EC_50_, 6.0 µM; **164**: EC_50_, 5.2 µM) activities.

Next, the same group studied the SARs for compound **158** further. This led to the discovery of compound **165** with high PL^pro^ inhibitory activity of SARS‐CoV‐1 (IC_50_, 0.32 µM) and antiviral activity (EC_50_, 9.1 µM) in Vero cells.^199^ The mode of action of **165** was found to be a noncovalent, competitive inhibition of PL^pro^. Unlike the previous series, the stereochemistry at the *α*‐methyl group did not make a significant difference in inhibition of PL^pro^. For example, both (*S*)‐ and (*R*)‐methyl inhibitors, **165** (IC_50_, 0.32 µM; EC_50_, 9.1 µM and **166** (IC_50_, 0.56 µM; EC_50_, 9.1 µM), respectively, shared equipotent inhibitory activity in enzymatic and cell‐based assays.

Further SARs of **159** and **165** were investigated to improve the activity. However, no significant improvement in the activity was observed for the prepared compounds either in the enzymatic or cell‐based bioassay. Compounds **167**–**169** (Figure 36) displayed the best inhibitory activities. Especially, the *m*‐fluoro‐substituted benzamide derivative **168** (IC_50_, 0.15 µM; EC_50_, 5.4 µM) showed the best inhibition activity against PL^pro^. It also inhibited SARS‐CoV‐1 in the cell‐based bioassay. Both compounds **168** and **169** were metabolically more stable when compared to **167**.

**Figure 37:**
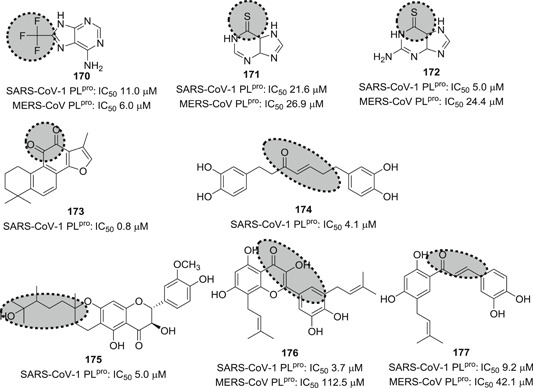
Broad spectral PL^pro^ inhibitors from different sources. PL^pro^, papain‐like protease; SARS‐CoV, severe acute respiratory syndrome coronavirus

HTS of a chemical library of 25000 molecules identified **170** (Figure 37) as a dual SARS‐CoV‐1 PL^pro^ (IC_50_, 10.9 µM) and MERS‐CoV PL^pro^ (IC_50_, 6.2 µM) inhibitor.^200^ This compound acts via competitive inhibition against MERS‐CoV PL^pro^, yet via allosteric inhibition against SARS‐CoV‐1 PL^pro^. This compound also exhibited a preference for SARS‐CoV‐1 PL^pro^ and MERS‐CoV PL^pro^ versus two human homologs of the PL^pro^, ubiquitin C‐terminal hydrolase, (hUCH‐L1) and (hUCH‐L3).

Chou et al.^201^ identified thiopurine (**171**) and 6‐thioguanine (**172**) as SARS‐CoV‐1 PL^pro^ inhibitors by the screening of a library containing 160 compounds. The thiocarbonyl group was important for PL^pro^ inhibition. However, the toxicity of these anticancer agents limits their therapeutic utility as anti‐SARS agents.

In 2012, Park et al.^202^ reported a tanshinone derivative **173** as a SARS‐CoV‐1 PL^pro^ inhibitor with an IC_50_ value of 0.8 µM. The same research group also described diarylheptanoids blocking SARS‐CoV‐1 PL^pro^. In particular compound **174** performed as the best inhibitor of SARS‐CoV PL^pro^ with an IC_50_ value of 4.1 µM. An α,β‐unsaturated carbonyl functionality was crucial for effective inhibition. The geranylated flavonoid **175** was another plant‐derived natural product, which displayed SARS‐CoV‐1 PL^pro^ inhibition with an IC_50_ value of 5.0 µM.^203^

In 2017, Park et al.^204^ assessed the inhibitory activity of polyphenols isolated from *B. Papyrifera* against SARS‐CoV PL^pro^ and MERS‐CoV PL^pro^. Two of them (**176** and **177** Figure 37) displayed moderate inhibition at both SARS‐CoV‐1 PL^pro^ and MERS‐CoV PL^pro^ with a noncompetitive mechanism of action.

Disulfiram (**151**; Figure 35) was also reported as a SARS‐CoV‐1 PL^pro^ inhibitor (IC_50_, 24.1 µM),^205^ probably by reacting with the active site cysteine, thereby covalently modifying the enzyme target, as was reported for other targets.

## 4 RdRP AND ITS INHIBITORS

The ability to produce new RNA copies from available template molecules is necessary for life on earth. RNA polymerases are therefore found in all living cells as well as many viruses. RdRP are essential enzymes to all RNA viruses, as they catalyze the synthesis of new RNA from a given RNA template.^206^ Due to their importance for viral life cycles, and their high conservation among different RNA viruses, they have been attractive drug targets for antiviral therapy for a long time.

SARS‐CoV‐2 also uses an RdRP to replicate its genome within the host cell. Three nonstructural viral proteins (nsp) form its replication/transcription complex, with nsp12 forming the catalytic subunit. Bound to it are nsp7 and nsp8—accessory factors that facilitate template binding.^207^, ^208^, ^209^ Their individual structures and that of the complex have been solved.^210^, ^211^, ^212^, ^213^, ^214^ Interestingly, the only nsp that interacts directly with RNA seems to be nsp12, whereas nsp7 and nsp8 are needed to increase its efficiency.^210^, ^215^

**Figure 38:**
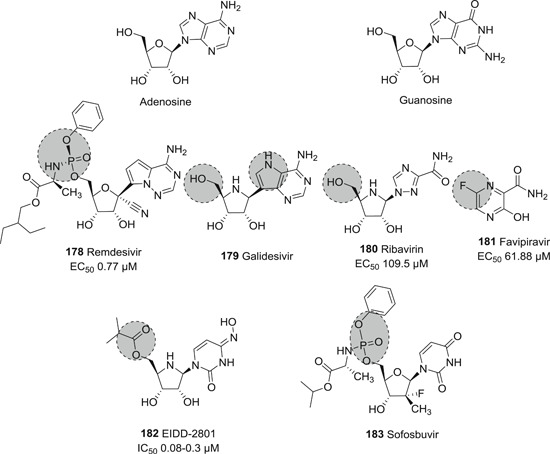
Nucleoside analogs with inhibition activity against SARS‐CoV‐2 RdRP (adenosine, guanosine, and sofosbuvir are included for comparison). RdRP, RNA‐dependent RNA polymerase; SARS‐CoV, severe acute respiratory syndrome coronavirus

RdRP is the target of inhibitors like remdesivir (**178**), galidesivir (**179**), ribavirin (**180**), favipiravir (**181**), and EIDD‐2801 (**182**). These molecules have shown promise for the treatment of COVID‐19 patients.^87^, ^216^, ^217^, ^218^ (For structures and biological data see Figure 38)

Remdesivir is a 1'‐cyano‐substitued adenine C‐nucleoside analog prodrug. The prodrug strategy used is similar to that of the FDA‐approved anti‐hepatitis C drug sofosbuvir (**183**; see Figure 38). Upon its diffusion into cells, the phosphoramidate undergoes an intracellular conversion process that results in the formation of the triphosphate active metabolite (RTP). The triphosphate is recognized as adenine by viral RdRP, which causes heavy disruptions in RNA synthesis.

The exact molecular mechanism of remdesivir's action against SARS‐CoV‐2 has recently been elucidated by Yin et al.^219^ who reported cryo‐EM structures of SARS‐CoV‐2 RdRP with remdesivir monophosphate (RMP) covalently bound to the primer strand. As only a single RMP was incorporated in each observed primer strand the inhibition mechanism was shown to be nonobligate RNA chain termination. The addition of RTP led to a complete inhibition of RNA polymerization activity at a concentration of 1 mM, even in the presence of ATP in high concentrations of 100 mM. The authors further highlight the high conservation level of catalytic sites of RdRPs in different RNA viruses, which makes the discovery of future broad‐spectrum antiviral RdRP inhibitors seem likely.

Remdesivir was previously reported to inhibit SARS‐CoV‐1 and MERS‐CoV replication in multiple in vitro systems, with submicromolar IC_50_ values.^220^ In primary human airway epithelial (HAE) cell cultures, the antiviral activity assessment of remdesivir against SARS‐CoV‐1 and MERS‐CoV showed a dose‐dependent reduction in replication with average IC_50_ values of 0.069 µM (SARS‐CoV‐1) and 0.074 µM (MERS‐CoV). In a mouse model of SARS‐CoV‐1 pathogenesis, remdesivir greatly decreased the virus concentration in the lung and mitigated clinical symptoms of infection and restored respiratory function.

Galidesivir is another C‐nucleoside analog that resembles adenosine. However, the base is not linked to a ribose, but to an aza‐sugar. Although it is recognized as adenosine by RdRP, its properties are different enough to cause a disruption in chain elongation. Galidesivir has been used in the treatment of Ebola and Marburg virus infections, and in vitro studies against SARS‐ and MERS‐CoVs have suggested efficacy against CoVs.^221^ Therefore, it is a likely future anti SARS‐CoV‐2 agent, and currently being studied in clinical trials.^222^, ^223^

Ribavirin is a nucleoside analog, which shows structural similarity to guanosine. But guanosine's 6‐membered ring is only hinted at by the amide group. As such, it is incorporated by viral RdRPs, but interrupts RNA polymerization.^224^ It is an approved drug in most countries and used against a variety of viral infections. Although its efficacy against SARS‐CoV‐2 has not been determined in large clinical trials, ribavirin has shown some promise in the treatment of COVID‐19 patients.^225^

Favipiravir (Avigan®) is an approved antiviral drug for the treatment of influenza in Japan and China. It is a pyrazinamide derivative that has shown some activity against a variety of RNA viruses.^226^ Favipiravir inhibits viral RdRP via its similarity to guanine. After biotransformation into its active metabolite, favipiravir‐ribofuranosyl‐5'‐triphosphate, it is incorporated into newly synthesized RNA by RdRP, leading to premature chain termination^227^ similar to remdesivir's mode of action. Favipiravir is currently being studied around the world as a treatment option against COVID‐19.

Very recently, Sheahan et al.^228^ reported the discovery of EIDD‐1931 and its orally bioavailable prodrug EIDD‐2801. These nucleoside analogs have shown remarkable potency against SARS‐CoV‐2 and other related CoVs in vitro and in vivo, with IC_50_ values in the low nanomolar range, outperforming remdesivir 3–10‐fold. The reason for this increased potency could be additional interactions with viral RdRP involving the N4‐hydroxyl group of the cytidine ring.^219^ The efficacy of EIDD‐2801 in COVID‐19 patients is being evaluated in a clinical trial.^229^

Remdesivir and other potential RdRP inhibitors^230^ are currently being studied in clinical trials around the world, but even though preliminary results appear promising, it is too early to assess their clinical value against COVID‐19.

## 5 DRUGS REPOSITIONING APPROACH

Drug repurposing is an attractive strategy for finding new indications for already well‐established, marketed drugs or highly characterized compounds. It is a fast way to identify of new therapeutic options directly available for clinical use or eligible for accelerated approval for various diseases and disorders. An extensive effort has been made in repurposing approved drugs since the outbreak of SARS‐CoV‐1. Below, we summarize selected drug repositioning strategies for anticoronaviral therapy and their results.

The host's innate interferon (IFN) response is one key for controlling viral replication. The IFN response can be increased by administering artificial IFNs and IFN inducers. The recombinant IFN‐α and ‐β inhibited the replication of SARS‐ and MERS‐CoVs in animal models.^231^ Several studies also described the combination of IFNs with antiviral drugs like ribavirin (**180**) or lopinavir‐ritonavir for treating SARS.^232^, ^233^

In 2004, SARS patients in an open‐label study had better clinical outcomes when treated with ribavirin in combination with lopinavir‐ritonavir (400 and 100 mg, respectively) than the control group receiving only ribavirin.^227^ A study in SARS patients found that viral replication could not be blocked at ribavirin concentrations achievable in human serum.^234^ Nevertheless, the combination of ribavirin with IFN‐β had a synergistic effect on the inhibition of SARS‐CoV‐1 replication. The effects of PEGylated IFN together with ribavirin against SARS‐CoV‐2 are being studied in clinical trials.^87^

**Figure 39:**
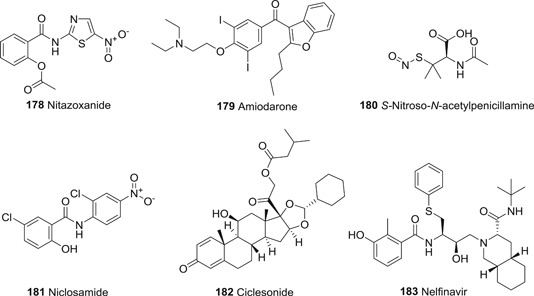
Selected structures of drugs suitable for repositioning against SARS‐CoV‐1 and 2. SARS‐CoV, severe acute respiratory syndrome coronavirus

Nitazoxanide (**178**; Figure 39), a broad‐spectrum antiparasitic drug, was reported to inhibit SARS‐CoV‐2 (EC_50_, 2.12 μM in Vero E6 cells).^215^ It is also an IFN‐inducing agent, and it is being studied for treating a wide range of infections.

The antiarrhythmic drug amiodarone (**179**, Figure 39) also inhibited SARS‐CoV‐1 replication in infected Vero cells.^235^ The drug appears to alter the endocytotic pathway, thus inhibiting endosomal viral entry.

Glycyrrhizin inhibited viral replication in Vero cells with an EC_50_ value of 300 mg/L, possibly by blocking viral entry as well.^232^

As nitric oxide (NO) has been associated with antiviral activity, the NO donor, *S*‐nitroso‐*N*‐acetylpenicillamine (**180**; Figure 39) was reported to inhibit SARS‐CoV‐1 replication in a dose‐dependent manner.^236^

In a search for potential antiviral agents against SARS‐CoV‐1, the screening of a library of 8000 approved drugs identified cinanserin (**150**; Figure 39), a serotonin antagonist, as a potential inhibitor of SARS‐CoV‐1 targeting its M^pro^ with IC_50_ value 4.0 µM.^189^

A virtual screening and docking study identified the calmodulin antagonist calmidazolium as a SARS‐CoV‐1 M^pro^ inhibitor (*K*_i_, 61 µM).^237^

In 2014, Dyall et al. reported an array of pharmaceutical drugs with antiviral activity against MERS‐CoV, and SARS‐CoV‐1 (Table 2, chemical structure of all drugs were indicated in Figure S1).^121^ The agents were grouped according to their modes of action. Hits inhibited both investigated CoVs.

**Table 2:** Compounds with inhibitory activity at MERS‐CoV and SARS‐CoV‐1

Drugs	Class	MERS‐CoVEC_50_ (µM)	SARS‐CoV‐1EC_50_ (µM)
Emetine	Antibacterial agent	0.014	0.051
Chloroquine	Antiparasitic agent	6.27	6.53
Hydroxychloroquine	Antiparasitic agent	8.27	7.96
Mefloquine	Antiparasitic agent	7.41	15.55
Amodiaquine	Antiparasitic agent	6.21	1.27
Loperamide	Antidiarrheal agent	4.8	5.90
Lopinavir	HIV‐1 inhibitor	8.0	24.4
E‐64‐D	Cathepsin inhibitor	1.27	0.76
Gemcitabine	DNA metabolism inhibitor	1.21	4.95
Tamoxifen	Estrogen receptor inhibitor	10.11	92.88
Toremifene	Estrogen receptor inhibitor	12.91	11.96
Terconazole	Sterol metabolism inhibitor	12.20	15.32
Triparanol	Sterol metabolism inhibitor	5.28	‐
Anisomycin	Protein‐processing inhibitor	0.003	0.19
Cycloheximide	Protein‐processing inhibitor	0.189	0.04
Homoharringtonine	Protein‐processing inhibitor	0.071	‐
Benztropine	Neurotransmitter inhibitor	16.62	21.61
Fluspirilene	Neurotransmitter inhibitor	7.47	5.96
Thiothixene	Neurotransmitter inhibitor	9.29	5.31
Chlorpromazine	Neurotransmitter inhibitor	9.51	12.97
Fluphenazine	Neurotransmitter inhibitor	5.86	21.43
Promethazine	Neurotransmitter inhibitor	11.80	7.54
Astemizole	Neurotransmitter inhibitor	4.88	5.59
Chlorphenoxamine	Neurotransmitter inhibitor	12.64	20.03
Thiethylperazine	Neurotransmitter inhibitor	7.86	‐
Triflupromazine	Neurotransmitter inhibitor	5.75	6.39
Clomipramine	Neurotransmitter inhibitor	9.33	13.23
Imatinib	Kinase signaling inhibitor	17.68	9.82
Dasatinib	Kinase signaling inhibitor	5.46	2.10

In particular, the protein‐processing inhibitors cycloheximide and anisomycin showed strong inhibitory activities against both CoVs. The HIV protease inhibitor lopinavir was more effective against SARS‐CoV‐1 than against MERS‐CoV. The antidiarrheal agent loperamide showed moderate inhibitory activitiy against both CoVs. The anti‐protozoal and emetic alkaloid with antibacterial properties, emetine, showed strong antiviral activity against MERS‐CoV. The antiparasitic drugs chloroquine, hydroxychloroquine, and mefloquine showed moderate antiviral activities against both CoVs. Cathepsin inhibitor, E‐64‐D, inhibited both as well. Two of the neurotransmitter inhibitors, chlorpromazine and triflupromazine also blocked both viruses (see Section 2) The DNA synthesis inhibitor gemcitabine was able to inhibit SARS‐CoV‐1 and MERS‐CoV with an EC_50_ value of 1.2 and 4.9 µM, respectively. Toremifene is an estrogen receptor 1 antagonist that inhibited both MERS‐CoV and SARS‐CoV‐1 (EC_50_, 12.9 and 11.97 µM, respectively).

Kinase signaling pathway inhibitors imatinib and dasatinib were active against both MERS‐CoV and SARS‐CoV‐1. Imatinib was reported to act at an early stage of viral infection by hampering the fusion of viral particles with the endosome.^53^

Niclosamide (**181**; Figure 39), an anthelmintic drug, exhibited very potent antiviral activity against SARS‐CoV‐1 replication and stopped viral antigen synthesis at 1.56 μM concentrations.^238^ It prevented the cytopathic effect of SARS‐CoV‐1 at low concentrations of 1 μM and halted SARS‐CoV‐1 replication with an EC_50_ less than 0.1 μM in Vero E6 cells.^188^ Gassen et al. demonstrated that niclosamide inhibits SKP2 activity, increases the lysine‐48‐linked polyubiquitination of the Benclin 1 level, boosts autophagy, and effectively impedes MERS‐CoV replication.^239^ Niclosamide inhibited MERS‐CoV replication by up to 1000‐fold at 48 h p.i. at 10 μM.^239^

Jeon et al. conducted a screening of FDA approved drugs in Vero cells to discover promising antiviral drug candidates against SARS‐CoV‐2 infection.^240^ They reported 24 drugs that exhibited antiviral efficacy with IC_50_ values between 0.1 and 10 µM.

Among them two approved drugs, niclosamide (**181**) and ciclesonide (**182**; Figure 39), exhibited notable inhibitory activities against virus replication in Vero cells. Niclosamide exhibited very potent antiviral activity against SARS‐CoV‐2 (IC_50_, 0.28 µM). The action of niclosamide might be attributed to autophagy as it was reported for MERS‐CoV.^239^ Ciclesonide (**182**; Figure 39) is another interesting drug candidate with far lower antiviral potency (IC_50_, 4.33 µM) compared to niclosamide. It is a cortisol derivative used to treat asthma and allergic rhinitis.^241^ A recent report by Matsuyama et al. confirmed ciclesonide as a possible antiviral drug against SARS‐CoV‐2.^242^ A treatment report of three COVID‐19 patients (https://www3.nhk.or.jp/nhkworld/en/news/20200303_20/) merits further clinical investigation of this drug. The molecular target of ciclesonide's antiviral activity was revealed to be NSP15, a viral riboendonuclease. Together with its well‐established anti‐inflammatory effects, ciclesonide could offer an interesting option for the control of COVID‐19 symptoms.

Azithromycin showed a synergistic effect in combination with hydroxychloroquine in vitro against SARS‐CoV‐2 at realistic concentrations reachable in the human lung. Clinical trials with this antibiotic were initiated in New York on 24 March 2020.^243^ Very recently, however, the clinical benefit of the drug in COVID‐19 patients was called into question.^82^

Studies for colchicine as an anti‐SARS‐CoV‐2 agent are currently ongoing with the aim of curtailing inflammation and lung complications in mild COVID‐19 cases.^244^

Famotidine has been proposed as a therapeutic against COVID‐19, and a clinical trial is underway.^245^ It is used to treat peptic ulcers and gastroesophageal reflux disease, among others. Cimetidine is a similar drug and has also been suggested as a treatment for COVID‐19.

Dipyridamole was proposed as a treatment for COVID‐19 as well, and a clinical study is being conducted.^246^ It is a nucleoside transport and PDE3 inhibitor that prevents blood clot formation.

Sildenafil was proposed as treatment for COVID‐19, and it is currently being investigated in a small trial.^247^ It is a medication used to treat erectile dysfunction and pulmonary arterial hypertension.

Fenofibrate and bezafibrate have been suggested for the treatment of COVID‐19. Fenofibrate is a blood lipid‐lowering medicine of the fibrate class. ^248^, ^249^ Bezafibrate is a related lipid‐lowering agent.

The HIV‐protease inhibitor nelfinavir (**183**; Figure 39) strongly inhibited replication of SARS‐CoV‐1 in Vero cells with an EC_50_ value of 0.048 µM. It was suggested to exert its effect at the post‐entry step of SARS‐CoV‐1 infection.^250^ Recently, Yamamoto et al reported that nelfinavir also potently inhibited replication of SARS‐CoV‐2 among nine other Anti‐HIV drugs tested (IC_50_, 1.13 µM; CC_50_, 24.32 µM; SI = 21.52).^251^ The measured serum concentrations of nelfinavir were 3–6 times higher than the reported EC_50_ of this drug. This indicates that it is a promising drug candidate for the management of COVID‐19. Other drugs tested against SARS‐CoV‐2 replication were amprenavir (EC_50_, 31.32 µM; CC_50_ > 81 µM; SI > 2.59), darunavir (EC_50_, 46.41 µM; CC_50_ > 81 µM; SI > 1.75), and indinavir (EC_50_, 59.14 µM; CC_50_ > 81 µM; SI > 1.37). Tipranavir inhibited SARS‐CoV‐2 replication as well (EC_50_, 3.34 µM; CC_50_, 76.80 µM; SI = 5.76). Ritonavir (EC_50_, 8.63 µM; CC_50_, 74.11 µM, SI = 8.59), saquinavir (EC_50_, 8.83 µM; CC_50_, 44.43 µM; SI = 5.03), and atazanavir (EC_50_, 9.36 µM; CC_50_ > 81 µM; SI > 8.65) suppressed SARS‐CoV‐2 at less than 10 µM. Lopinavir, which was studied in SARS and COVID‐19 patients, also potently inhibited SARS‐CoV‐2 replication with the highest selectivity index (EC_50_, 5.73 µM; CC_50_, 74.44 µM; SI = 12.99).

**Figure 40:**
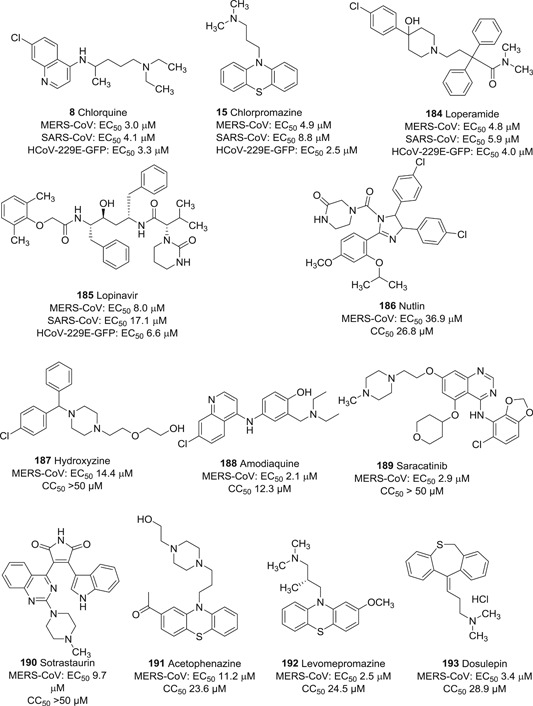
Drugs repurposed for MERS‐ and SARS‐CoV infections. MERS, Middle East respiratory syndrome; SARS‐CoV, severe acute respiratory syndrome coronavirus

De Wilde et al. identified four drugs—chloroquine (**8**), chlorpromazine (**15**), loperamide (**184**), and lopinavir (**185**)—by screening of an FDA approved drugs library (for structures, see Figure 40).^252^ All of them blocked SARS‐CoV‐1, MERS‐CoV, and HCoV‐229E replication at small concentrations, suggesting potential as broad‐spectrum virostatic agents.

Chloroquine (**8**) was able to inhibit SARS‐CoV‐2 viral replication in vitro, but a recent study found no clinical benefit for COVID‐19 patients who had received the drug^82^ (the drug is discussed in detail in the section *viral entry inhibitors*).

Chlorpromazine (**15**) stopped the replication of SARS‐CoV‐1, MERS‐CoV, and HCoV‐229E. It is a neuroleptic drug used against schizophrenia;^253^ here, it interferes with clathrin‐mediated endocytosis. Since, clathrin‐mediated endocytosis is a crucial port for viral entry into the host cell, used by MHV,^254^, ^255^, ^256^ SARS‐CoV‐1,^99^ and MERS‐CoV,^102^ future clinical trials could help elucidate this drug's therapeutic potential against COVID‐19.

Loperamide (**184**), an opioid receptor agonist against diarrhea,^257^ inhibited the replication of SARS‐CoV‐1, MERS‐CoV, and HCoV‐229E.

Lopinavir (**185**) is an HIV protease inhibitor and was previously shown to block SARS‐CoV‐1 M^pro^.^258^

Shin et al. analyzed a library of 2334 approved medications and bioactive molecules to find possible antiviral compounds against MERS‐CoV.^259^ A series of hit compounds was identified, categorized as anticancer (**189**, **190**), antipsychotics (**191**, **192**), and antidepressant (**193**) with inhibition activity between 2.1 and 14.4 µM (Figure 38). Saracatinib (**189**) was especially interesting, as it had remarkable anti‐MERS‐CoV activity (EC_50_ of 2.9 µM, CC_50_ > 50 µM). It is a small molecule drug with oral bioavailability used in the management of malignant neoplasms via Src‐family tyrosine kinases (SFKs) inhibition. It also suppressed other CoVs such as SARS‐CoV‐1 (EC_50_, 2.4 µM), HCoV‐229E (EC_50_, 5.1 µM), and FIPV (EC_50_, 7.0 µM) at nontoxic concentrations. Drugs **190** to **193** showed moderate antiviral activities.

## 6 Conclusions and future directions

The SARS‐CoV‐2 outbreak has caused worldwide disruption and was recently declared a global pandemic by the World Health Organization (WHO) owing to its rapid spread and high fatality rate. As there is no effective treatment to date, the number of infections continues to rise globally. This has led numerous research groups around the world to prioritize the identification and development of new therapeutics against COVID‐19.

Although it is often considered the most promising method to prevent or contain future coronavirus outbreaks, an all‐round anti‐CoV vaccine is possibly a long way away. Small molecule drugs have the potential to be effective, rapidly produced, and widely available. Indeed, several small molecules have been investigated and advanced to clinical trials for the treatment of COVID‐19, selected drug candidates are indicated in Table 3 (https://covid-19.heigit.org/clinical_trials.html).

**Table 3:** Selection of molecules investigated in ongoing clinical studies (July, 2020)

Name	Structure	Description	Recent trials
Azithromycin	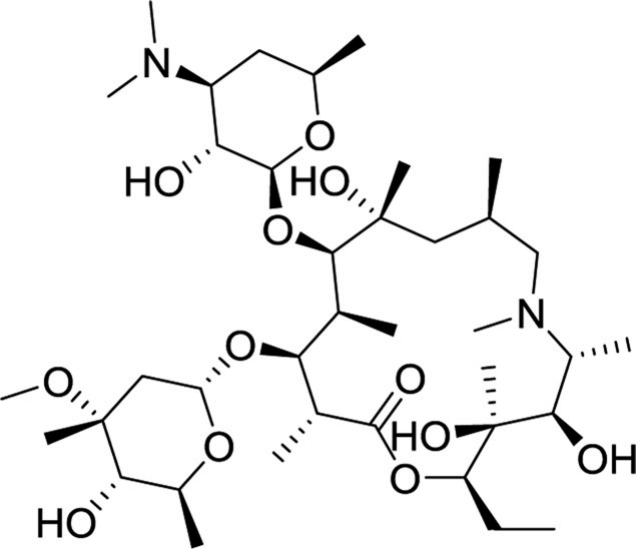	Macrolide antibiotic	IRCT20200428047228N2 NCT04405921 EUCTR2020‐001605‐23‐ES
Favipiravir	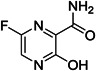	Broad spectrum antiviral drug	NCT04434248 ChiCTR2000033491 NCT04425460
Triazavirin	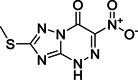	Broad‐spectrum antiviral drug	ChiCTR2000030001
Umifenovir	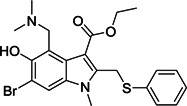	Antiviral drug	IRCT20200523047550N1 IRCT20151227025726N15 IRCT20200325046859N2
Baloxavir marboxil	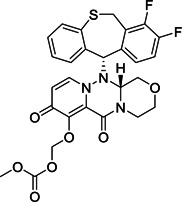	Antiviral drug	ChiCTR2000029548 ChiCTR2000029544
Remdesivir	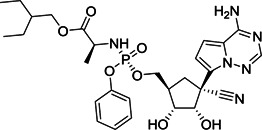	Antiviral drug	NCT04431453 NCT04409262 NCT04410354
Ribavirin	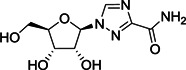	Antiviral drug	IRCT20200324046850N2 ChiCTR2000030922
Lopinavir/ritonavir	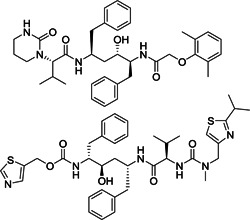	Anti‐HIV combination medication	NCT04403100 NCT04376814
Celecoxib	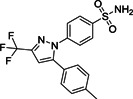	COX‐2 selective NSAID	ChiCTR2000031630
Chloroquine	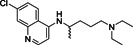	Antimalarial drug	EUCTR2020‐001441‐39‐IT NCT04447534 NCT04443270
Hydroxychloroquine	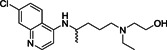	Antimalarial drug	EUCTR2020‐001558‐23‐IT EUCTR2020‐001441‐39‐IT EUCTR2020‐001501‐24‐IT
Mefloquine	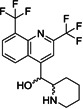	Antimalarial drug	EUCTR2020‐001194‐69‐ES
Ivermectin	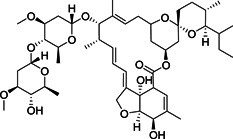	Broad‐spectrum antiparasitic drug	NCT04445311 NCT04435587 NCT04431466
Colchicine	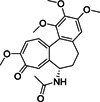	Broad‐spectrum anti‐inflammatory drug	NCT04416334 EUCTR2020‐001841‐38‐ES IRCT20190810044500N5
Corticosteroids (misc.)	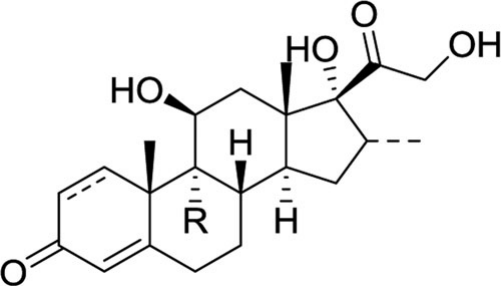	Broad‐spectrum anti‐inflammatory drug	IRCT20151227025726N17 NCT04395105 IRCT20120215009014N354
Pirfenidone	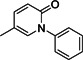	Antifibrotic, anti‐inflammatory drug	IRCT20200314046764N1 ChiCTR2000031138
Tranilast	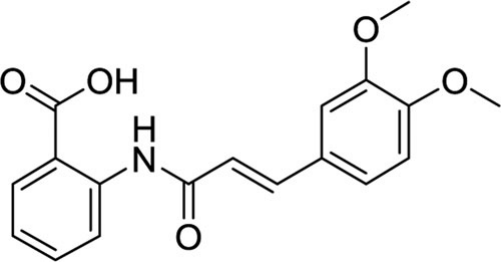	Anti‐inflammatory drug	ChiCTR2000030002
Selinexor	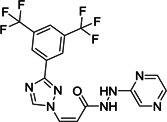	Anticancer drug	NCT04355676 NCT04349098 EUCTR2020‐001411‐25‐GB
Valsartan	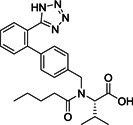	Angiotensin II receptor antagonist	DRKS00021732 NCT04335786
Dipyridamole	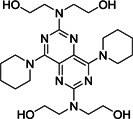	PDE3 inhibitor	NCT04424901 NCT04391179
Vidoflumidus (IMU‐838)	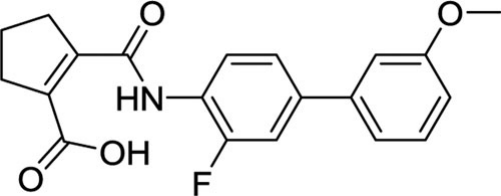	Investigational drug	EUCTR2020‐001264‐28‐HU
Azvudine	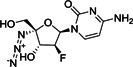	Investigational antiviral	ChiCTR2000032769 NCT04425772 ChiCTR2000030487
EIDD‐2801	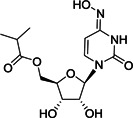	Investigational antiviral	NCT04405570 NCT04405739 NCT04392219

As outlined in this review, inhibitors of important viral enzymes or structures, such as M^pro^, PL^pro^, or RdRP have displayed encouraging activity against various human‐infecting CoVs. Since, both contagious viruses, SARS‐CoV‐1 and SARS‐CoV‐2, have a similar mechanism of infection; and both share the same human receptor, ACE2, for viral entry, for example—already developed inhibitors against the former could potentially be used to combat the latter. But despite the efficacy demonstrated by many inhibitors of SARS‐CoV‐1, no specific prophylactic or postexposure therapy is currently available.

The first step in the viral life cycle is the viral entry. It represents an attractive intervention point by blocking the RBD‐ACE2 interaction or the virus‐cell membrane fusion event. A large number of inhibitors, including peptides, antibodies, small‐molecule compounds, and natural products have been identified to hamper viral entry. Some of the peptides and antibodies displayed substantial anti‐SARS activity and are therefore considered promising entry inhibitors with high potencies in the low micromolar range.

Despite the apparent match of SARS‐CoV‐2 S and ACE2, other possible viral entry receptors should not be left unexplored. The glucose‐regulated protein 78 (GRP‐78, aka HSPA5), for instance, is employed as a coreceptor for entry by several viruses, including bat‐CoVs and MERS‐CoV,^260^ and a study predicted that SARS‐CoV‐2 S might utilize this mechanism as well.^261^ Elevated levels of GRP‐78 in COVID‐19 patients suggest a supplementary link.^262^ Although as of yet unconfirmed, the development of therapeutics against additional targets like GRP‐78 should receive due attention.

Viral proteases are another very important target for the development of antiviral therapies, as they are directly involved in the viral replication processes. Especially the M^pro^ is one of the best‐characterized viral targets, and numerous medicinal chemistry efforts have been already reported for the past outbreaks of SARS‐1 and MERS. Main proteases are highly conserved among other CoVs, which allows the development of broad spectral antiviral agents. Moreover, no human protease analog to the M^pro^ is known. Thus, drugs targeting M^pro^ could be highly virus‐selective and safe.

In light of the urgency of the current outbreak, repositioning of already approved drugs is becoming a popular approach due to the availability of toxicity and safety data. Drug repurposing has become fashionable, promising quick solutions to complicated questions. Old and, presumably, safe drugs are proclaimed miracle cures. The reality is a different one: Widely employed broad‐spectrum antiviral drugs, such as (hydroxy)chloroquine, favipiravir, ribavirin, or umifenovir were reported to be effective against SARS‐CoV‐2, but could not convince in clinical trials yet. Clinicians are faced with an avalanche of contraindications and a myriad of case reports to choose the right drug. The drug repositioning strategy is, therefore, not a sound scientific path to a cure. At best, it can provide a basis for extensive future research in all related fields, including synthetic organic medicinal chemistry.

A new problem with the current COVID‐19 outbreak is related to the spread of scientific information. When initial unfounded speculations about the alleged dangers of antihypertensive therapies with ACEis and ARBs were widely publicized in the media they caused great uncertainty among patients. Impetuous communications such as these can have serious consequences and should not be proclaimed carelessly. As it turns out, the benefits of continued antihypertensive therapy with these medicines in COVID‐19 patients far outweigh their risks. There is even evidence of additional protective effects of ACEis and ARBs in this cohort, although the clinical relevance of this has yet to be investigated.

It is clear that governments and societies all over the world have been surprised by the recent coronavirus outbreak—as they were by the SARS outbreak in 2003 and the MERS epidemic in 2013. Human‐infecting CoVs are on the rise, but quickly forgotten once life returns to normal. However, this problem will not disappear by itself, but likely increase in intensity. Viral spillover events are expected to increase in frequency as humans continue to invade new territories. We hope that, this time, the world will heed nature's warning to finance and conduct groundbreaking research on CoVs and their disease patterns. Only with a profound understanding of the viral life cycle and the affected human physiology we can prevent and control future outbreaks.

## CONFLICT OF INTERESTS

The authors declare there is no conflict of interest.

## AUTHORS CONTRIBUTIONS

TP, LLW, MM, and ME collected the data. TP wrote the manuscript, which was revised by all.

## Supporting information

Supporting information.
