# Supplementary material for: The recent outbreaks of human coronaviruses: A medicinal chemistry perspective
Source: Med Res Rev. 2020 Aug 27;41(1):72–135. doi: 10.1002/med.21724 (PMC7461420; doi:10.1002/med.21724)
Supplement: Supplementary file 1 — Supporting information. [file MED-41-72-s001.docx]

**Supporting information**

**The recent outbreaks of human coronaviruses: A medicinal chemistry perspective**

Thanigaimalai Pillaiyar,^a,†,*^ Lukas L. Wendt,^a,†^ Manoj Manickam,^b,†^ and Maheswaran Easwaran^c^

^a^PharmaCenter Bonn, Pharmaceutical Institute, Pharmaceutical & Medicinal Chemistry, University of Bonn, An der Immenburg 4, D-53121 Bonn, Germany

^b^Department of Chemistry, PSG Institute of Technology and Applied Research, Coimbatore, Tamil Nadu, India

^c^Department of Biomedical Engineering, Sethu Institute of Technology, Pulloor, Kariapatti, Virudhunagar 626115, Tamilnadu, India

^†^These authors contributed equally to this work.

^*^Corresponding author: Dr. Thanigaimalai Pillaiyar, e-Mail: [thanigai@uni-bonn.de](mailto:thanigai@uni-bonn.de)

Content

**Figure S1** Chemical structures of compounds from Table 2 Page 2-3

**Figure S1** Chemical structures of compounds from Table 2.
